# Data on nitrogen-containing derivatives of fumaropimaric acid

**DOI:** 10.1016/j.dib.2018.04.059

**Published:** 2018-04-25

**Authors:** T.B. Khlebnikova, V.N. Konev, Z.P. Pai

**Affiliations:** Boreskov Institute of Catalysis, Department of Catalytic Processes of Fine Chemical Synthesis, Akad. Lavrentiev Pr. 5, Novosibirsk 630090, Russia

## Abstract

The data presented here are related to the research paper entitled “Levopimaric Acid Derived 1,2-Diamines and Their Application in the Copper-Catalyzed Asymmetric Henry Reaction” [1]. In this data article, we provide ^1^H, ^13^C NMR and IR data for the diterpene derivatives described in [1]. The GC–MS analysis of pine oleoresin used as a starting material of the syntheses is also included in the data article.

**Specifications table**

**Table:** 

Subject area	*Chemistry*
More specific subject area	*Organic synthesis, natural products*
Type of data	*Synthetic schemes, NMR and IR spectra, GC-chromatogram*
How data was acquired	*NMR spectroscopy: Bruker DRX-500, АМ-400 and AV-300; IR spectroscopy: Infralum FT-801 and Shimadzu IRAffinity-1 FT-IR; GC–MS analysis: SHIMADZU GCMS-QP2010 Ultra instrument on the basis of gas chromatograph GC-2010 plus with mass detector*
Data format	*Raw, analyzed.*
Experimental factors	*The new diterpene derivatives were synthesized and purified by column chromatography or crystallization*
Experimental features	*The synthesized compounds were characterized by NMR and IR spectroscopy*
Data source location	*Novosibirsk, Russian Federation*
Data accessibility	*Data are available with this article*

**Value of the data**

• The data presents NMR, IR spectra of newly synthesized diterpene derivatives and GC–MS analysis of methylated pine oleoresin and could be used by other researchers.
• The provided information on the structural data of diterpenes could be useful for the analysis of spectra and determination of the structure of other diterpene derivatives.
• The data could be helpful for other researchers to identify the compounds described in the research article [1] and to reproduce the experiments reported therein.

## 1 Data

**Fig. 1:**
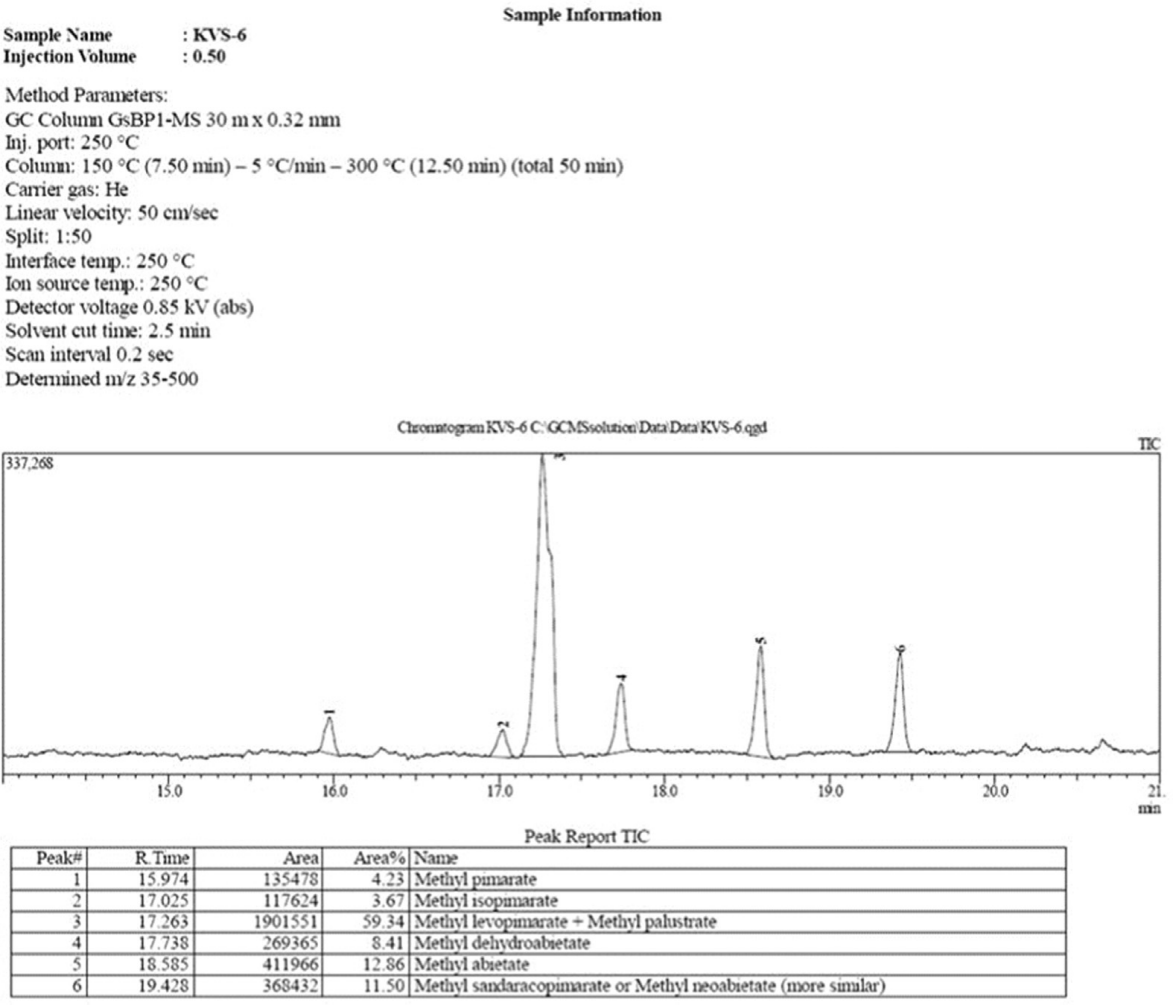
Chromatogram of methylated oleoresin (methyl esters of resin acids).

**Scheme 1:**
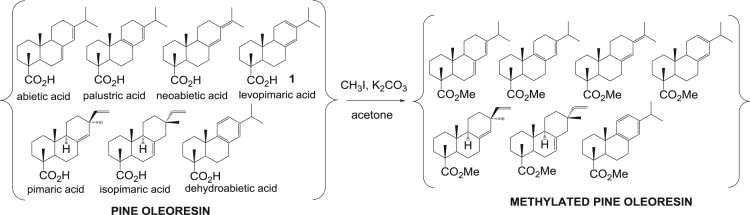
Methylation of resin acids.

**Scheme 2:**
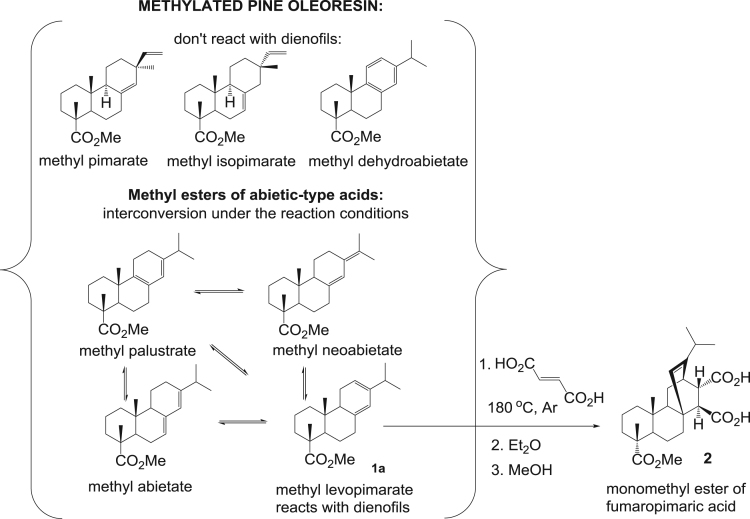
Synthesis of monomethyl ester of fumaropimaric acid **2** via Diels-Alder reaction of methyl levopimarate **1a** with fumaric acid.

**Scheme 3:**
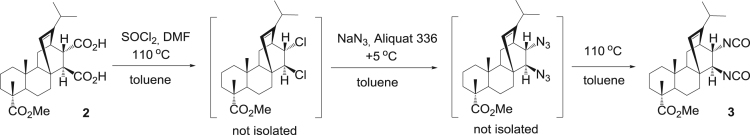
Synthesis of diisocyanate **3** from diacid **2** via Curtius rearrangement.

**Scheme 4:**
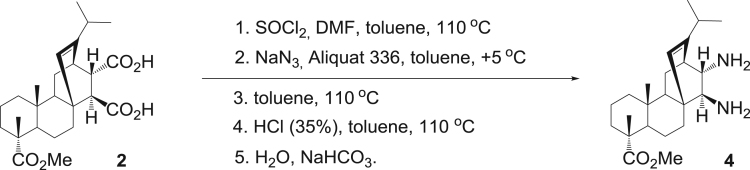
Synthesis of diamine **4** from diacid **2**.

**Scheme 5:**
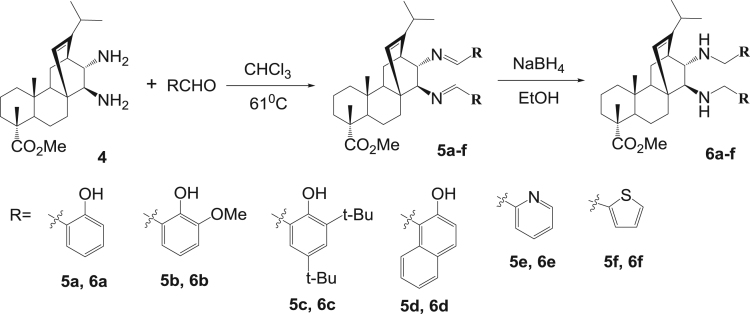
Synthesis of imines **5a-f** and aminophenols **6a-f**.

The dataset presented in this article focuses on characterization of the new diterpene derivatives described in [1]. The article provides the information on the composition of natural raw material (pine oleoresin) and the structural data of the functionalized diterpenes. Scheme 1 illustrates the preparation of mixture of methylated resin acids. The GC–MS analysis of this mixture is given in Fig. 1. Scheme 2 illustrates the method of preparation and isolation of monomethyl ester of fumaropimaric acid **2**. The compound **2** was characterized using ^1^H, ^13^C NMR and IR (Fig. 2-1, Fig. 2-2, Fig. 2-3). Scheme 3 illustrates the synthetic route to the 1,2-diisocyanate **3**, which was characterized using ^1^H, ^13^C NMR and IR (Fig. 3-1, Fig. 3-2, Fig. 3-3). Scheme 4 illustrates the synthetic route to the 1,2-diamine **4**, which was characterized using ^1^H, ^13^C NMR and IR (Fig. 4-1, Fig. 4-2, Fig. 4-3). Scheme 5 illustrates the method of preparation of imines **5a-f** and aminophenols **6a-f.** Figs. 5a-f and 6a-f shows ^1^H, ^13^C NMR and IR spectra of the compounds **5a-f** and **6a-f**. Analyses of the spectra of the compounds **2** and **4** are provided in [2]. Analyses of the spectra of the compounds **3**, **5a-f** and **6a-f** are provided in [1]. The synthetic procedures for the compounds **2**–**6** are described in the research article [1].

**Fig. 2-1:**
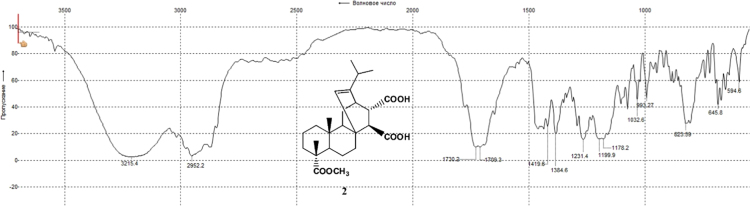
IR spectrum of compound **2**.

**Fig. 2-2:**
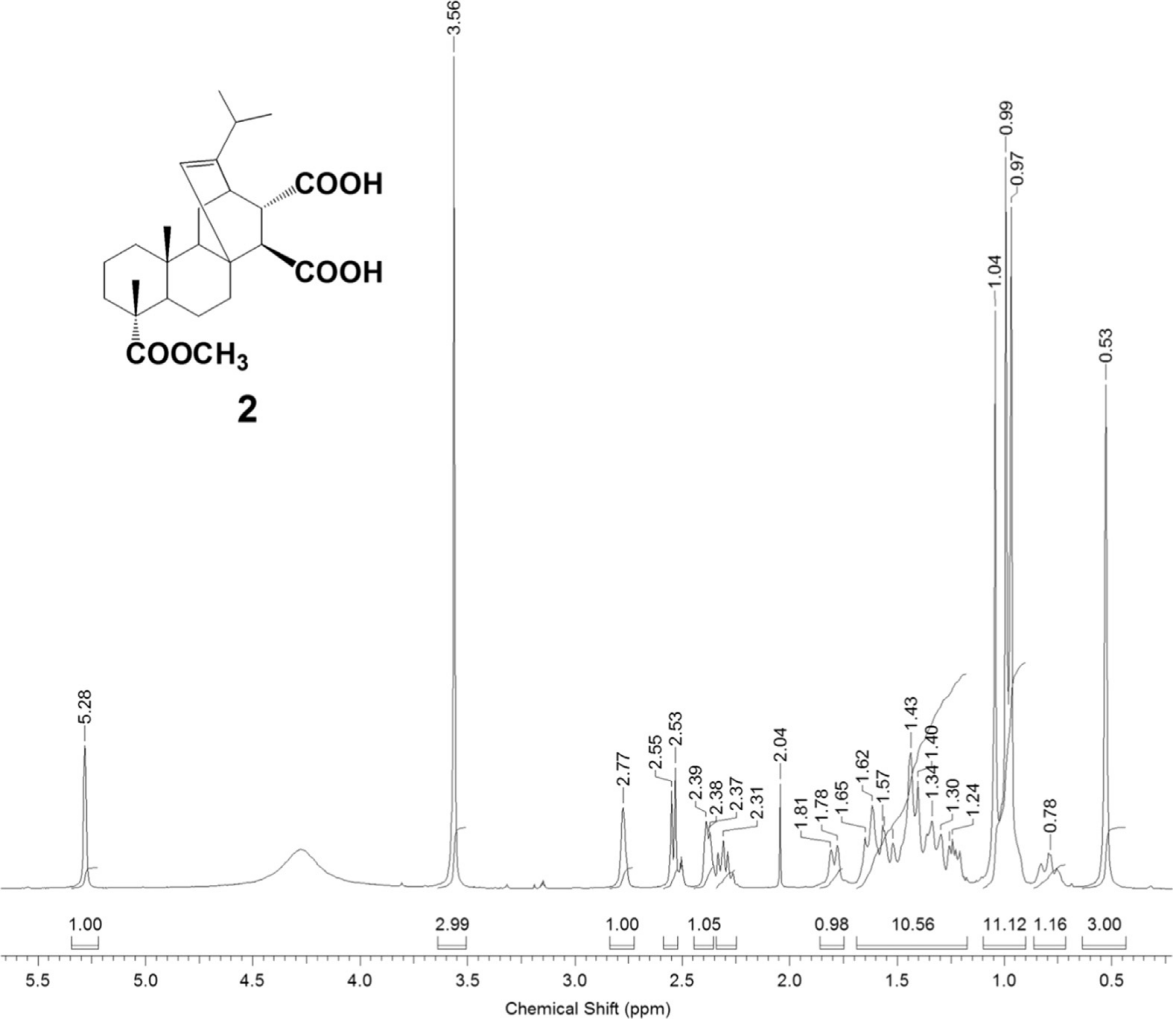
^1^H NMR spectrum of compound **2**.

**Fig. 2-3:**
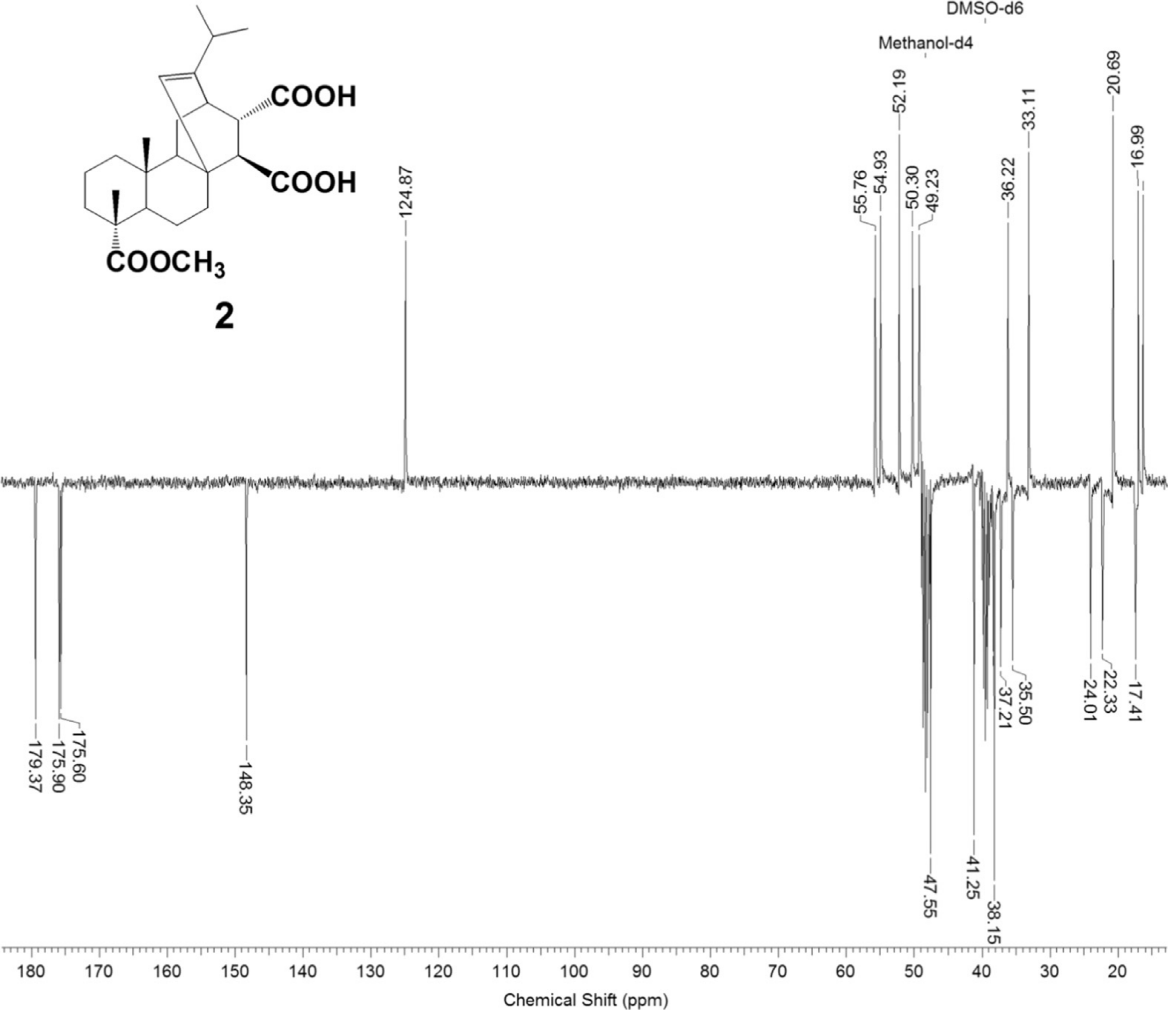
^13^C NMR spectrum of compound **2**.

**Fig. 3-1:**
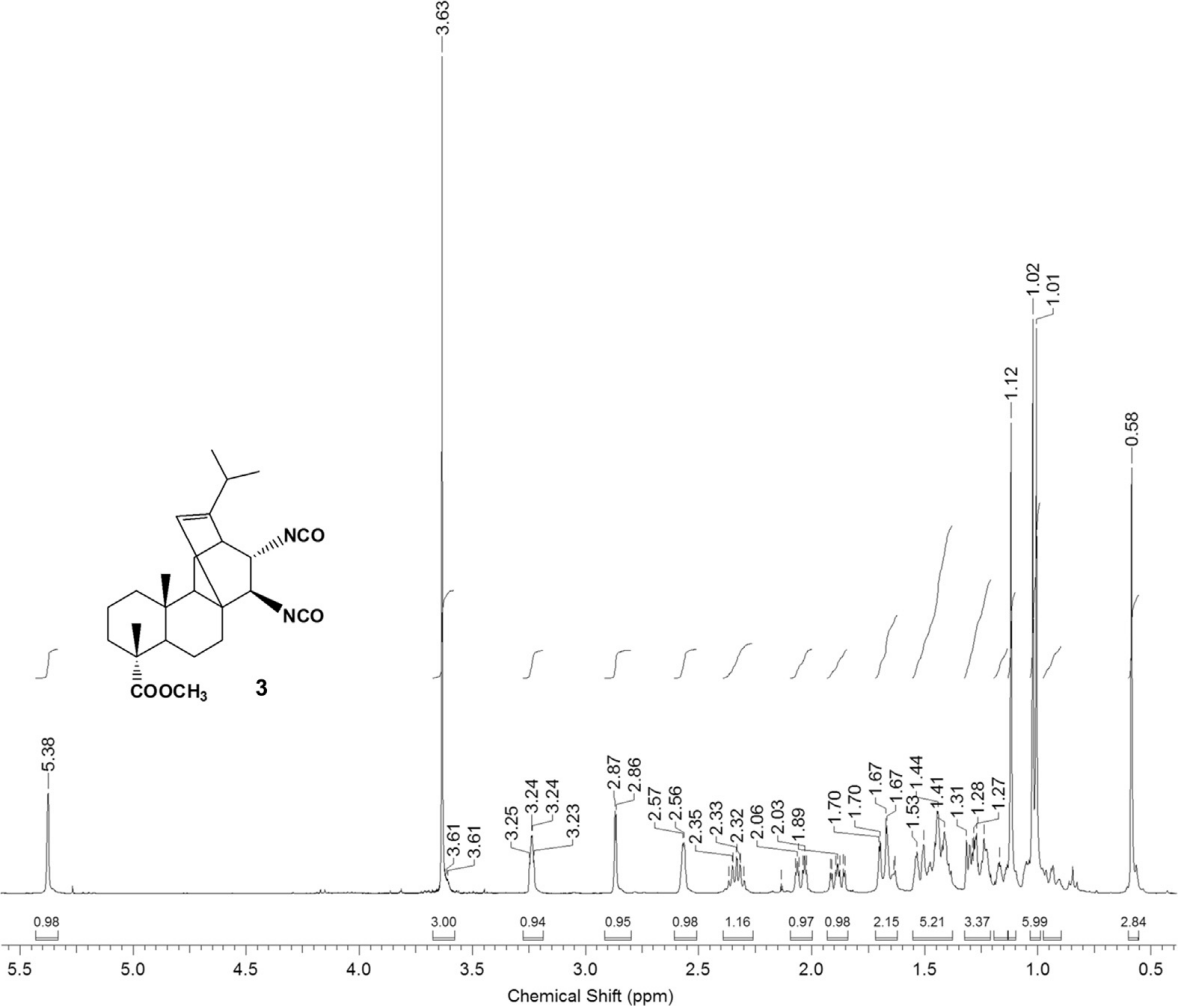
^1^H NMR spectrum of compound **3**.

**Fig. 3-2:**
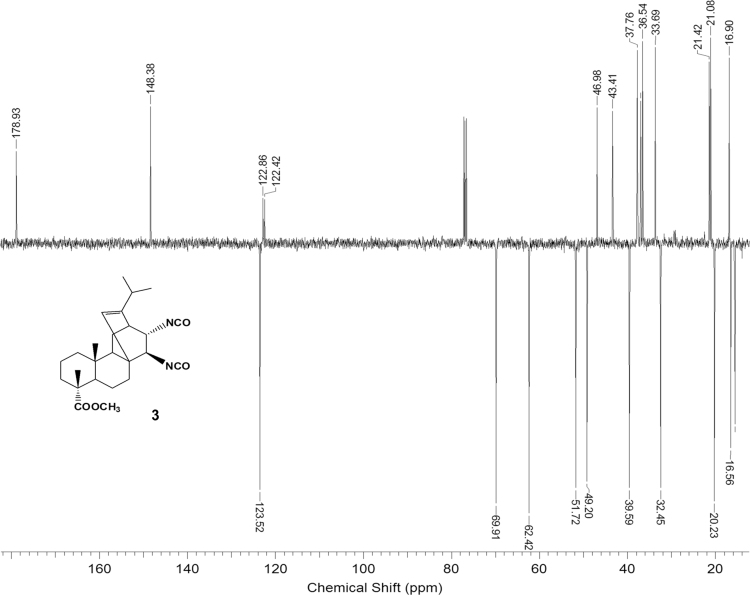
^13^C NMR spectrum of compound **3**.

**Fig. 3-3:**
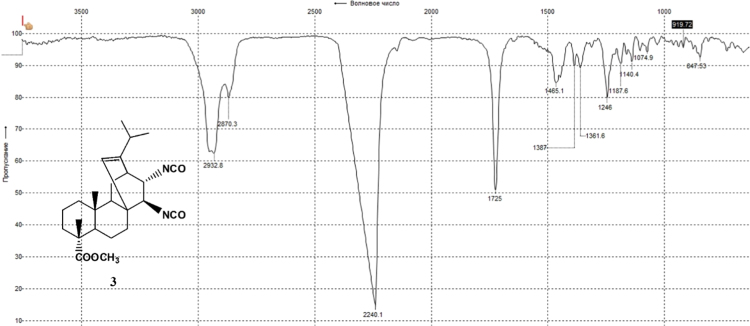
IR spectrum of compound **3**.

**Fig. 4-1:**
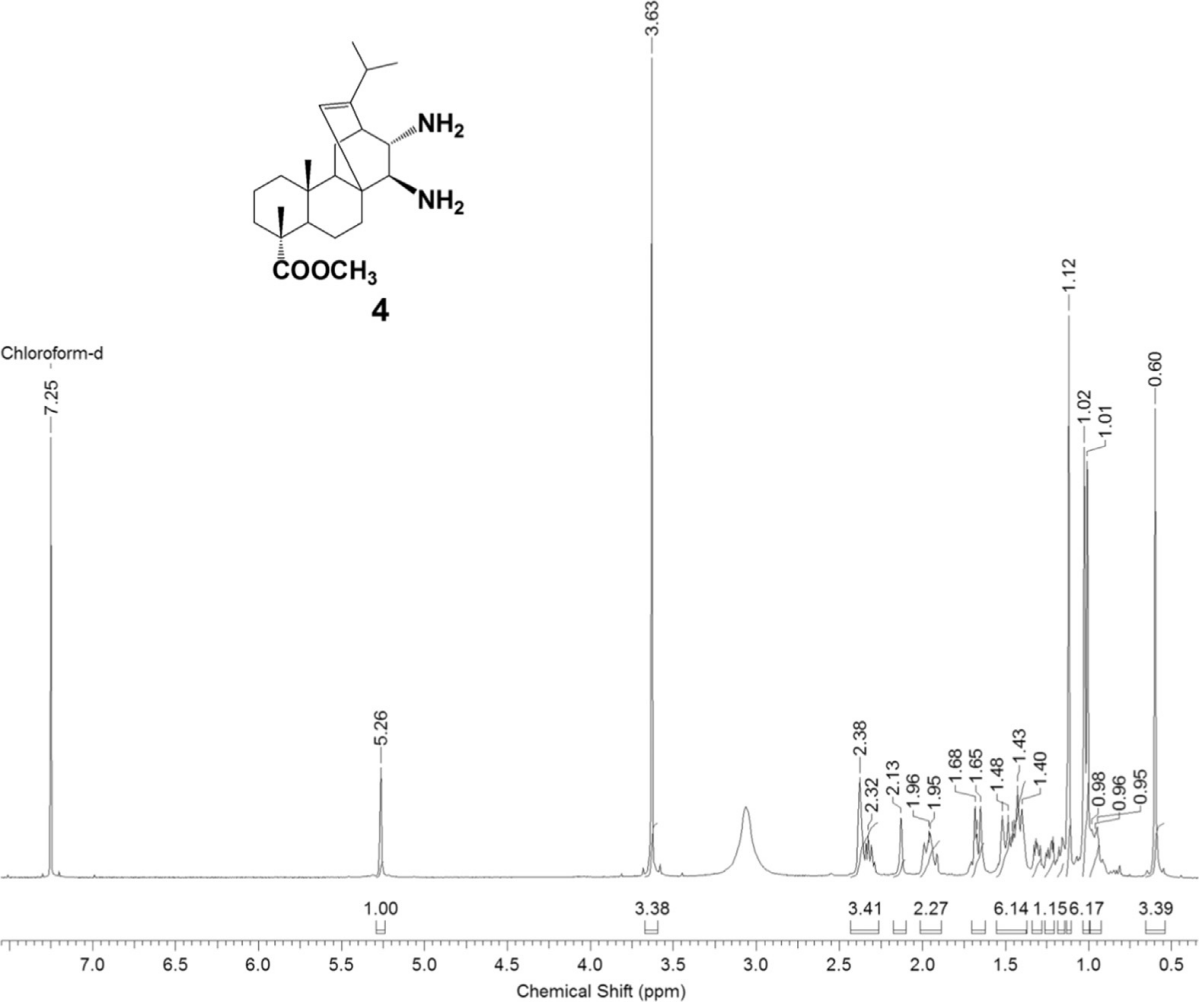
^1^H NMR spectrum of compound **4**.

**Fig. 4-2:**
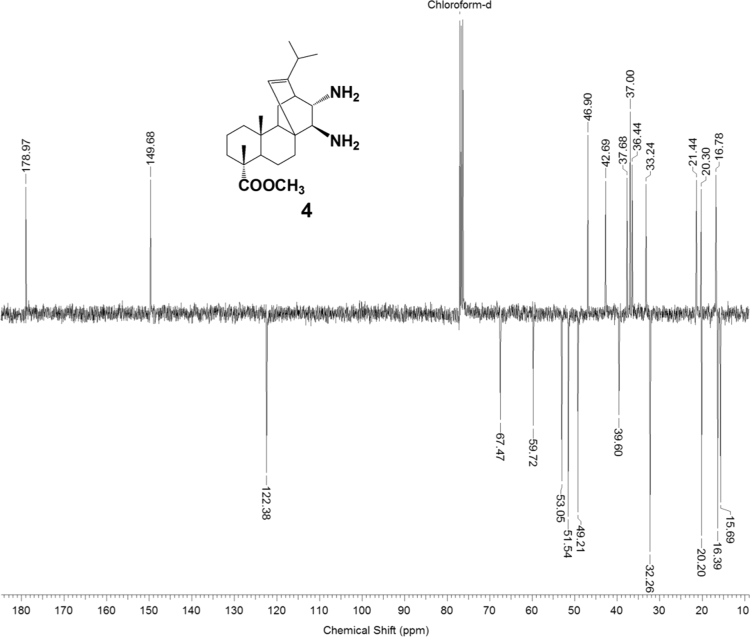
^13^C NMR spectrum of compound **4**.

**Fig. 4-3:**
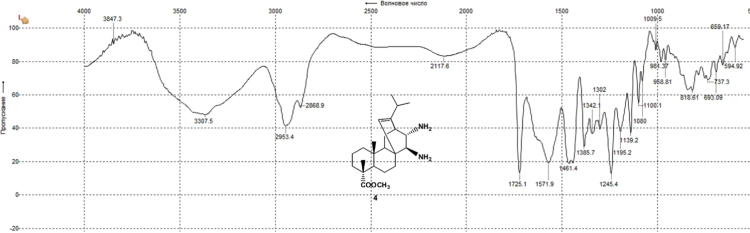
IR spectrum of compound **4**.

## 2 Experimental design, materials and methods

### 2.1 General information

The chemicals were of reagent purity grade, obtained from commercial sources, and used without further purification. Pine oleoresin OST 13-128-93 (Russian industry standard; oleoresin contains at least 80% of abietic-type acids) was obtained from Orgsyntez OJSC (available on request at http://orgsyntez.ru/en) and used as received. Solvents were distilled from appropriate drying agents prior to use, unless otherwise noted. Flash column chromatography was performed on silica gel (Panreac 40–63 µm). ^1^H NMR and ^13^C NMR spectra were recorded on Bruker DRX-500, АМ-400 and AV-300 spectrometers. Chemical shifts were reported in the δ scale using the residual solvent peak of the CHCl_3_ as a reference (726 ppm) for ^1^H NMR spectra and the middle signal in the triplet of CDCl_3_ (77.00 ppm) for ^13^C NMR samples. IR spectra were recorded using Infralum FT-801 or Shimadzu IRAffinity-1 FT-IR spectrometers. GC–MS analysis of methyl esters of resin acids was carried out on SHIMADZU GCMS-QP2010 Ultra instrument on the basis of gas chromatograph GC-2010 plus with mass detector and with chromatographic column GsBP1-MS 30 m × 0.32 mm.

## 3 Fumaropimaric acid derivatives

### 3.1 Methylated pine oleoresin

.

### 3.2 13-isopropyl-17,18-dinor-atis-13-ene-15β,16α-dicarboxy-4-carboxylic acid methyl ester 2

.

### 3.3 13-isopropyl-17,18-dinor-atis-13-ene-15β,16α-diisocyanato-4-carboxylic acid methyl ester 3

.

### 3.4 13-isopropyl-17,18-dinor-atis-13-ene-15β,16α-diamino-4-carboxylic acid methyl ester 4

.

### 3.5 Imines 5a-f

#### 3.5.1 13-Isopropyl-17,18-dinor-atis-13-ene-15β,16α-di(2-hydroxybenzylideneamino)-4-carboxylic acid methyl ester 5a

^1^H, ^13^C NMR and IR spectra of the compound **5a** are presented in Fig. 5a-1, Fig. 5a-2, Fig. 5a-3.

**Fig. 5a-1:**
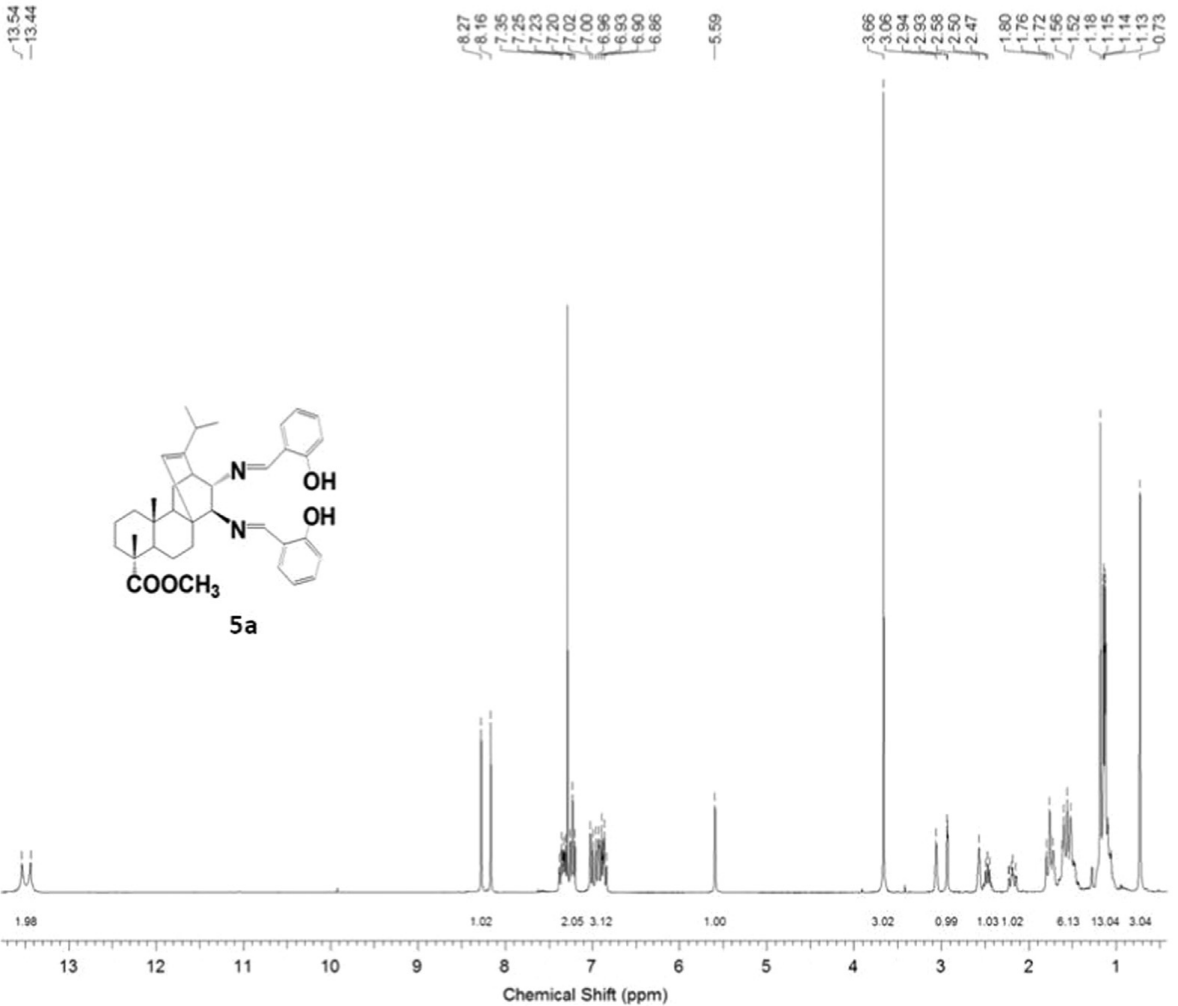
^1^H NMR spectrum of compound **5a**.

**Fig. 5a-2:**
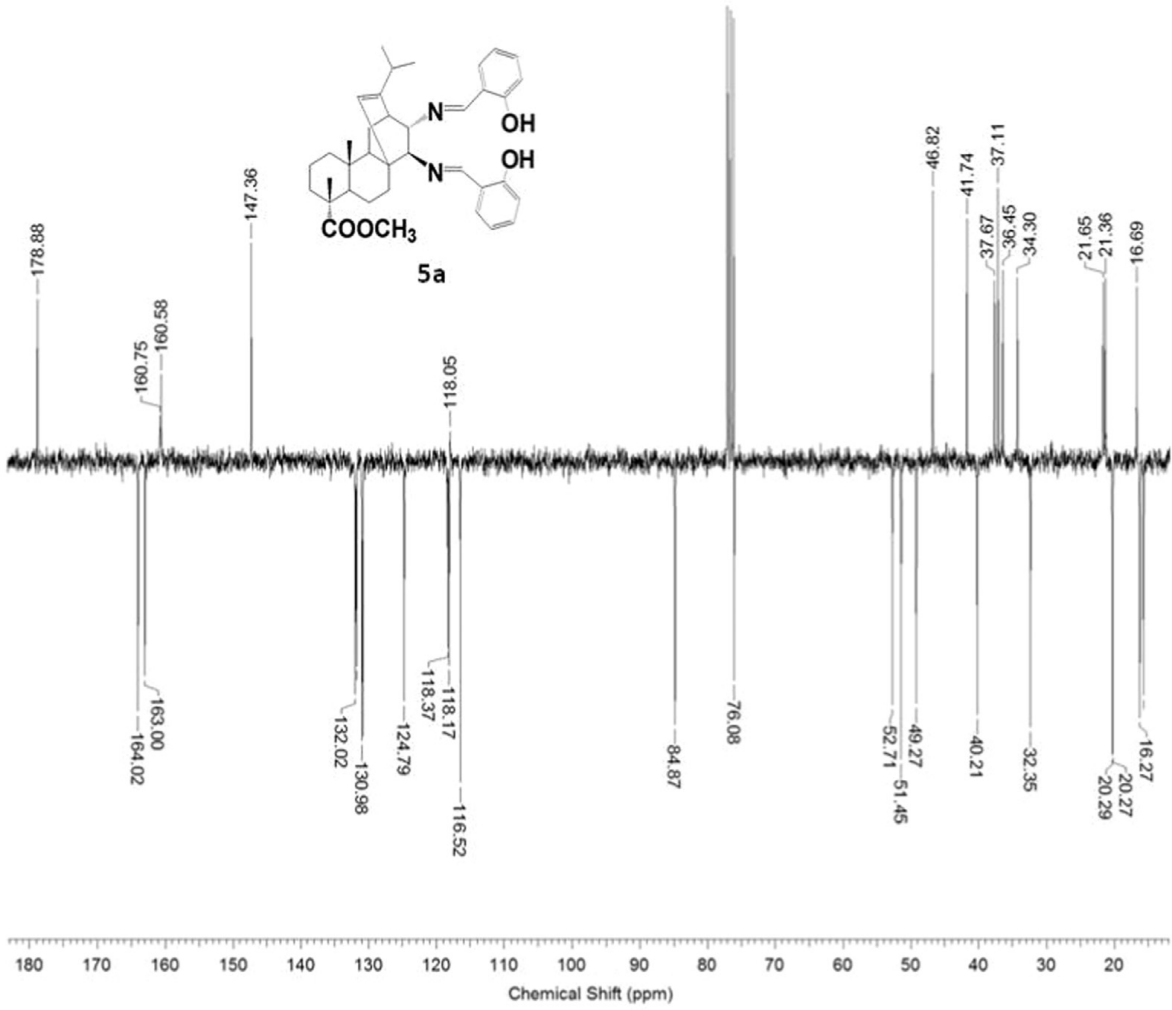
^13^C NMR spectrum of compound **5a**.

**Fig. 5a-3:**
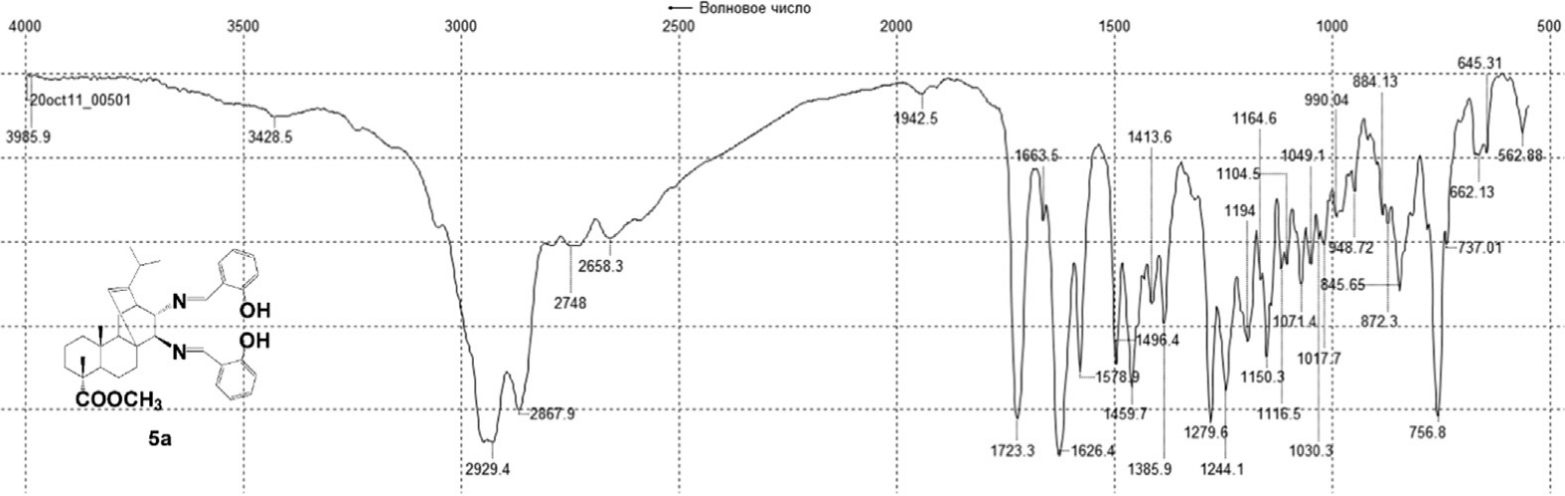
IR spectrum of compound **5a**.

#### 3.5.2 13-isopropyl-17,18-dinor-atis-13-ene-15β,16α-di(2-hydroxy-3-methoxybenzylideneamino)-4-carboxylic acid methyl ester 5b

^1^H, ^13^C NMR and IR spectra of the compound **5b** are presented in Fig. 5b-1, Fig. 5b-2, Fig. 5b-3.

**Fig. 5b-1:**
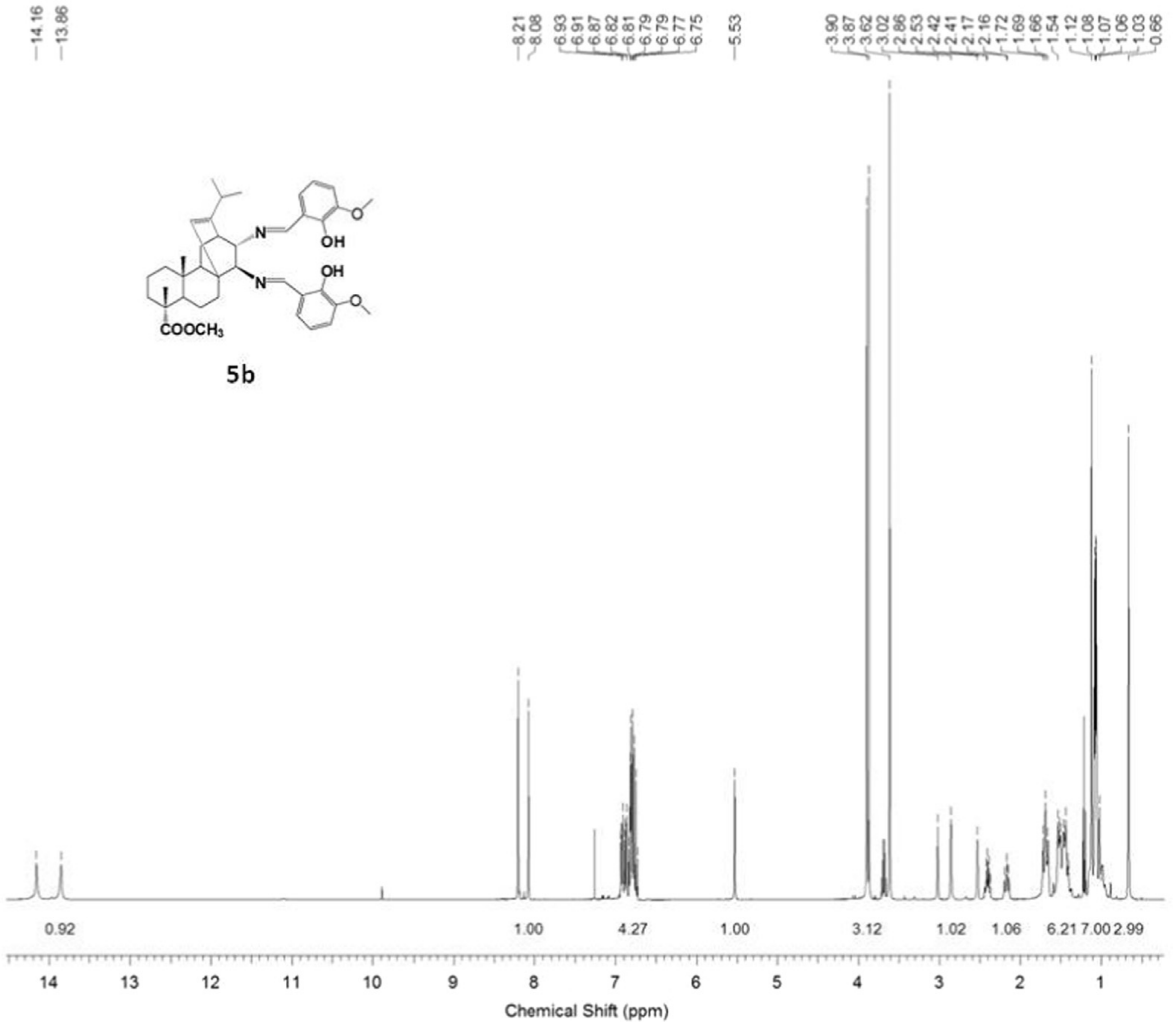
^1^H NMR spectrum of compound **5b**.

**Fig. 5b-2:**
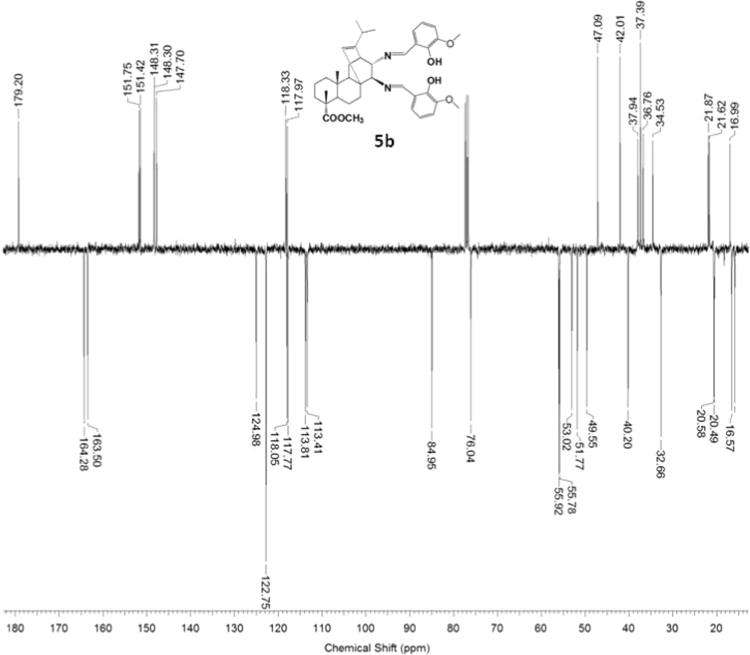
^13^C NMR spectrum of compound **5b**.

**Fig. 5b-3:**
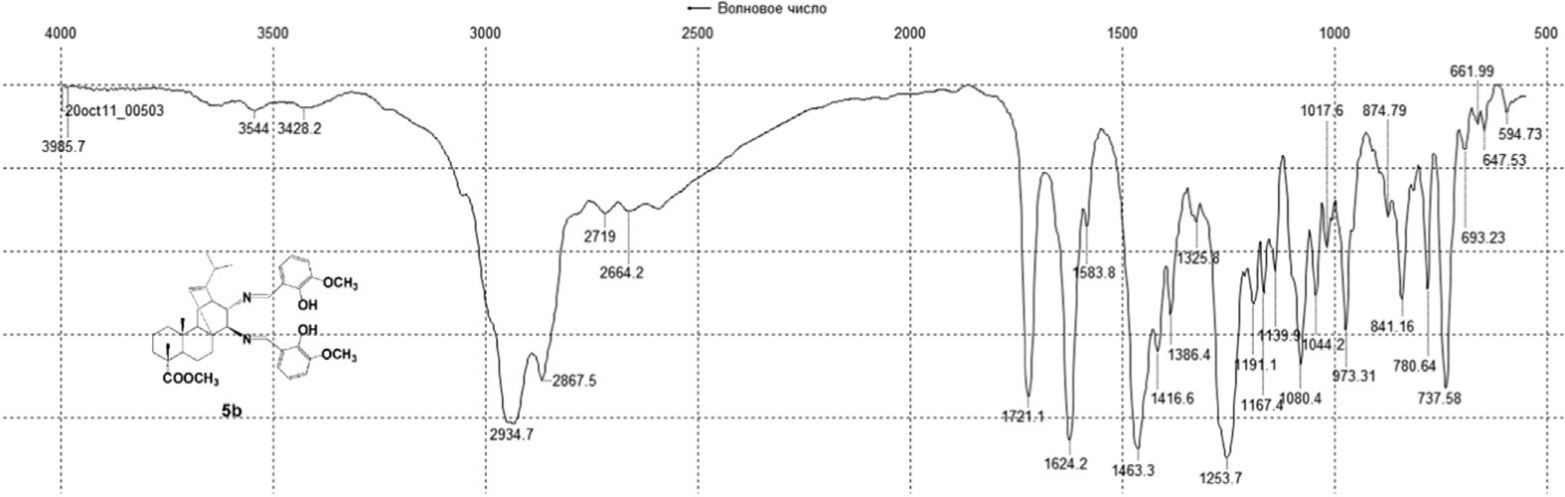
IR spectrum of compound **5b**.

#### 3.5.3 13-isopropyl-17,18-dinor-atis-13-ene-15β,16α-di(2-hydroxy-3,5-di-tert-butylbenzylideneamino)-4-carboxylic acid methyl ester 5c

^1^H, ^13^C NMR and IR spectra of the compound **5c** are presented in Fig. 5c-1, Fig. 5c-2, Fig. 5c-3.

**Fig. 5c-1:**
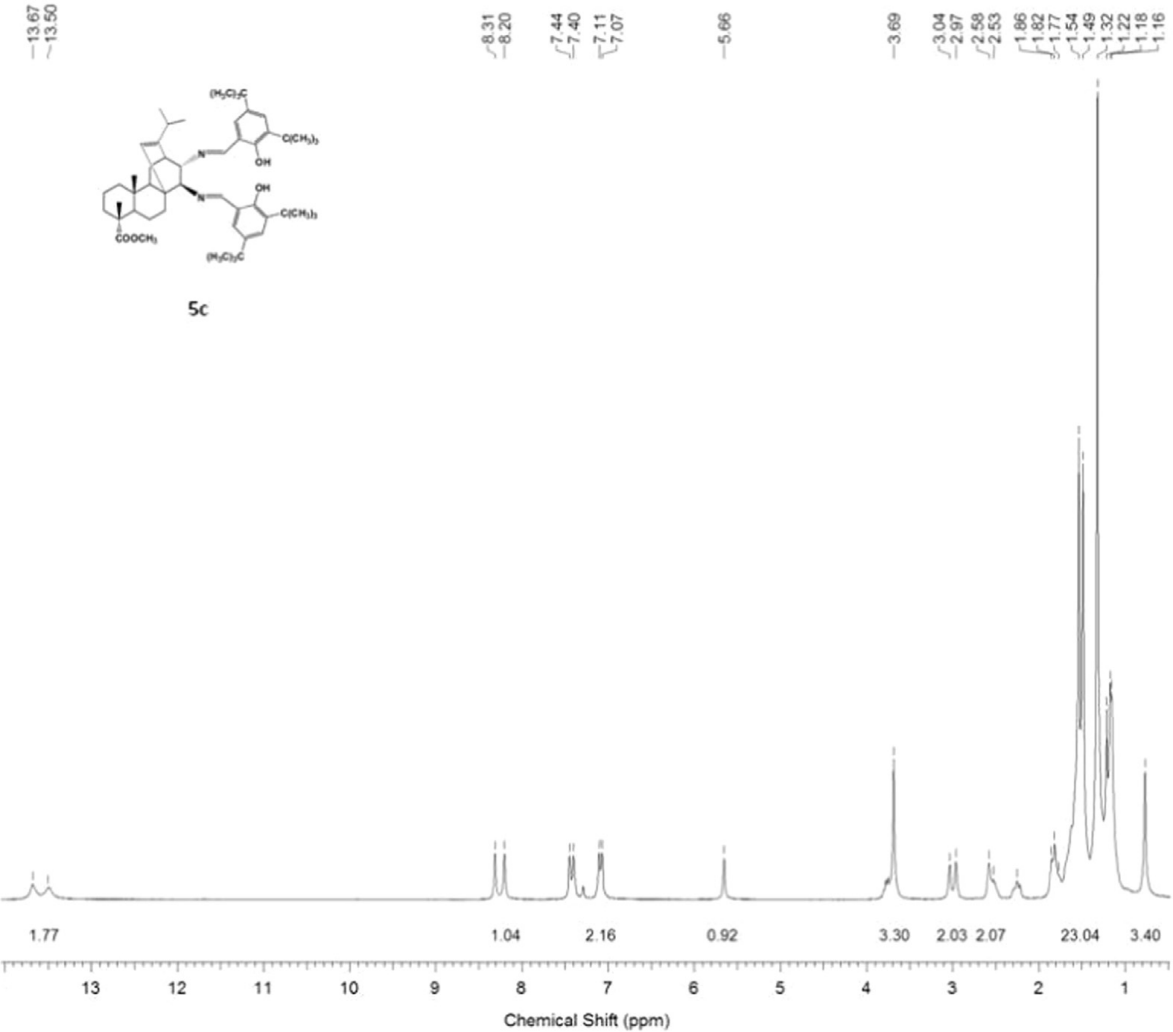
^1^H NMR spectrum of compound **5c**.

**Fig. 5c-2:**
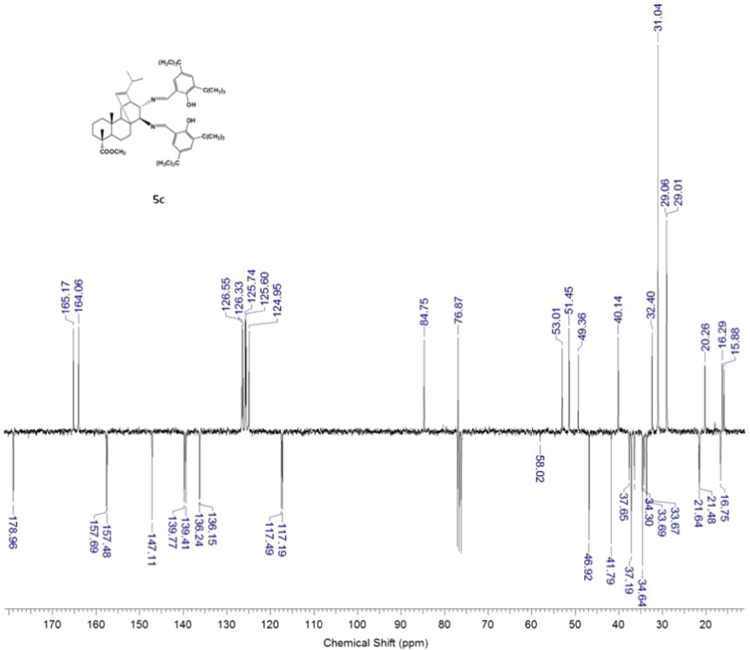
^13^C NMR spectrum of compound **5c**.

**Fig. 5c-3:**
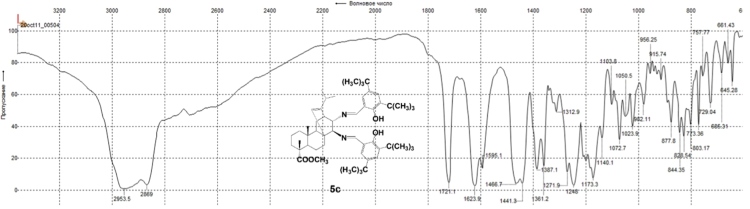
IR spectrum of compound **5c**.

#### 3.5.4 13-isopropyl-17,18-dinor-atis-13-ene-15β,16α-di(2-hydroxy-1-naphthylmethyleneamino)-4-carboxylic acid methyl ester 5d

^1^H, ^13^C NMR and IR spectra of the compound **5d** are presented in Fig. 5d-1, Fig. 5d-2, Fig. 5d-3..

**Fig. 5d-1:**
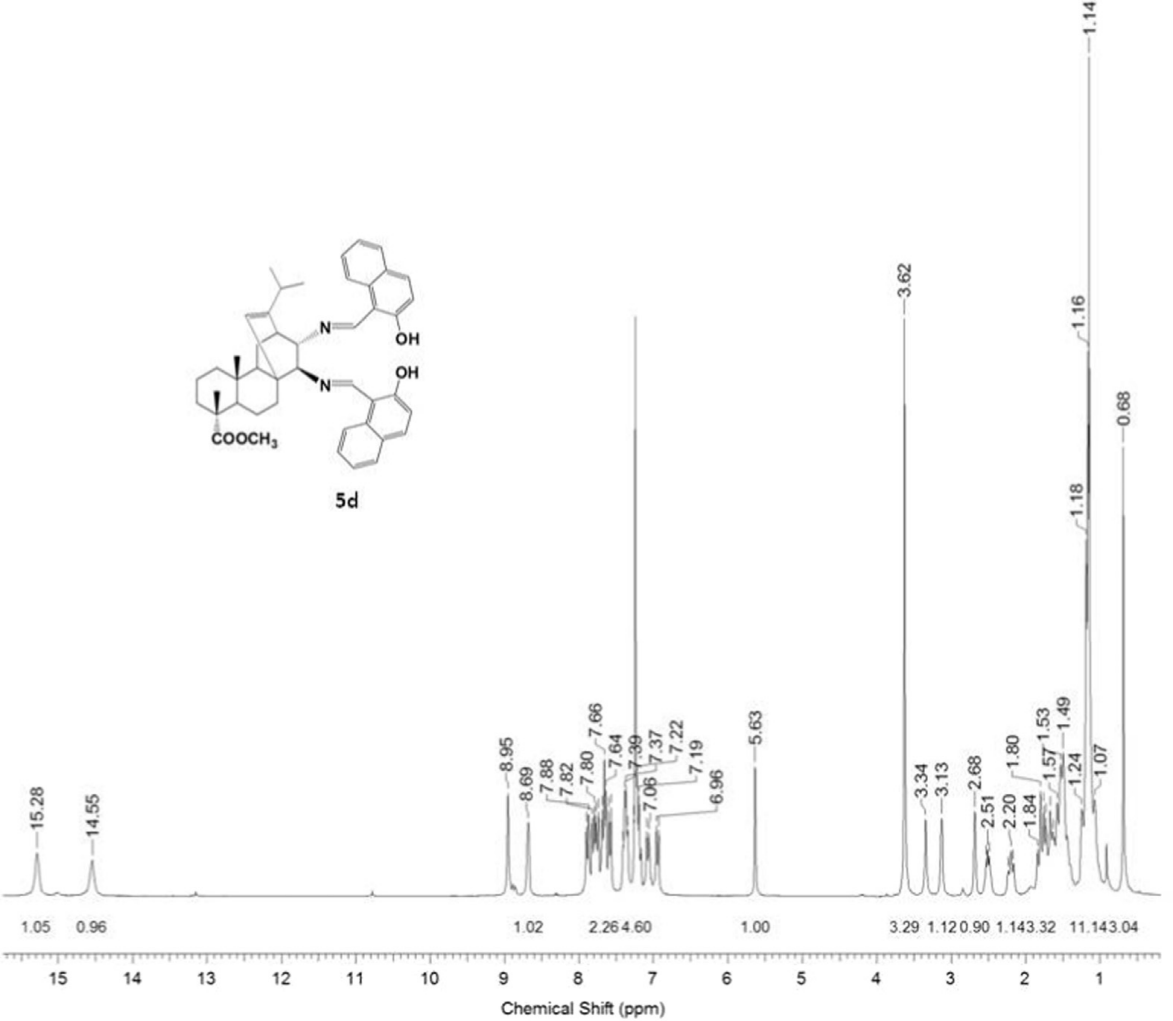
^1^H NMR spectrum of compound **5d**.

**Fig. 5d-2:**
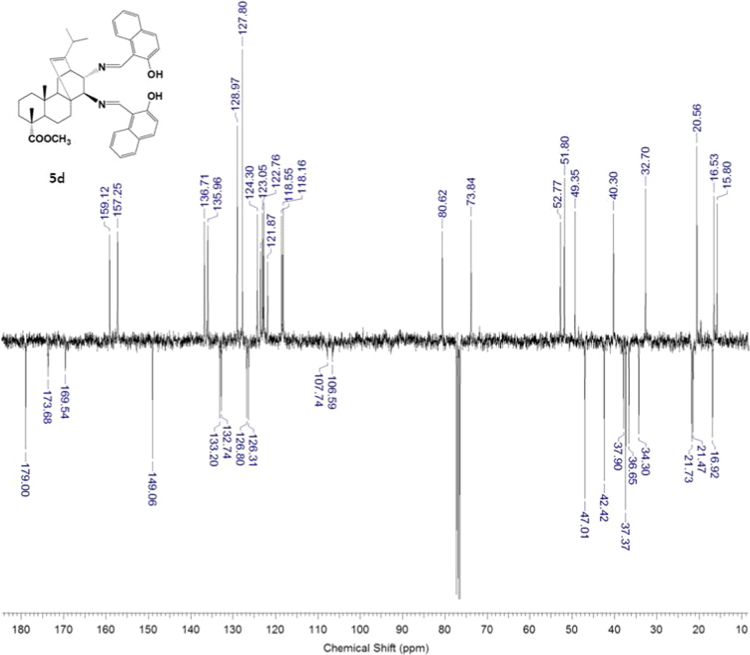
^13^C NMR spectrum of compound **5d**.

**Fig. 5d-3.**
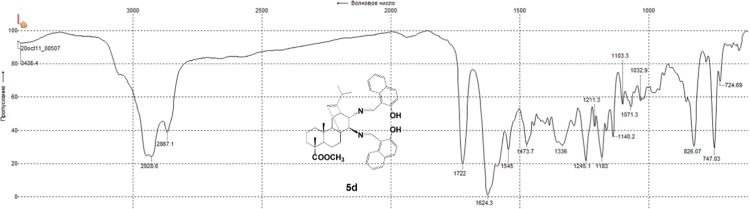
IR spectrum of compound **5d**.

#### 3.5.5 13-isopropyl-17,18-dinor-atis-13-ene-15β,16α-di(2'-pyridyl-methyleneamino)-4-carboxylic acid methyl ester 5e

^1^H, ^13^C NMR and IR spectra of the compound **5e** are presented in Fig. 5e-1, Fig. 5e-2, Fig. 5e-3.

**Fig. 5e-1:**
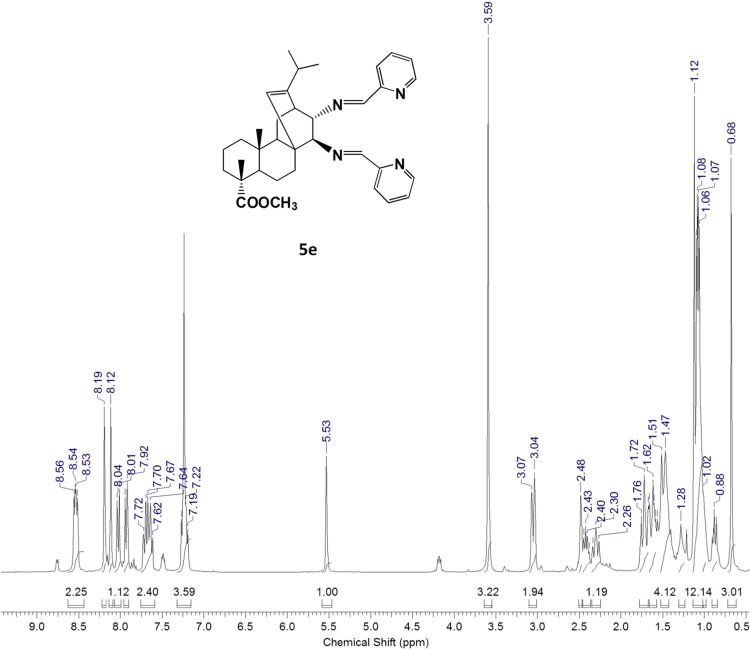
^1^H NMR spectrum of compound **5e**.

**Fig. 5e-2:**
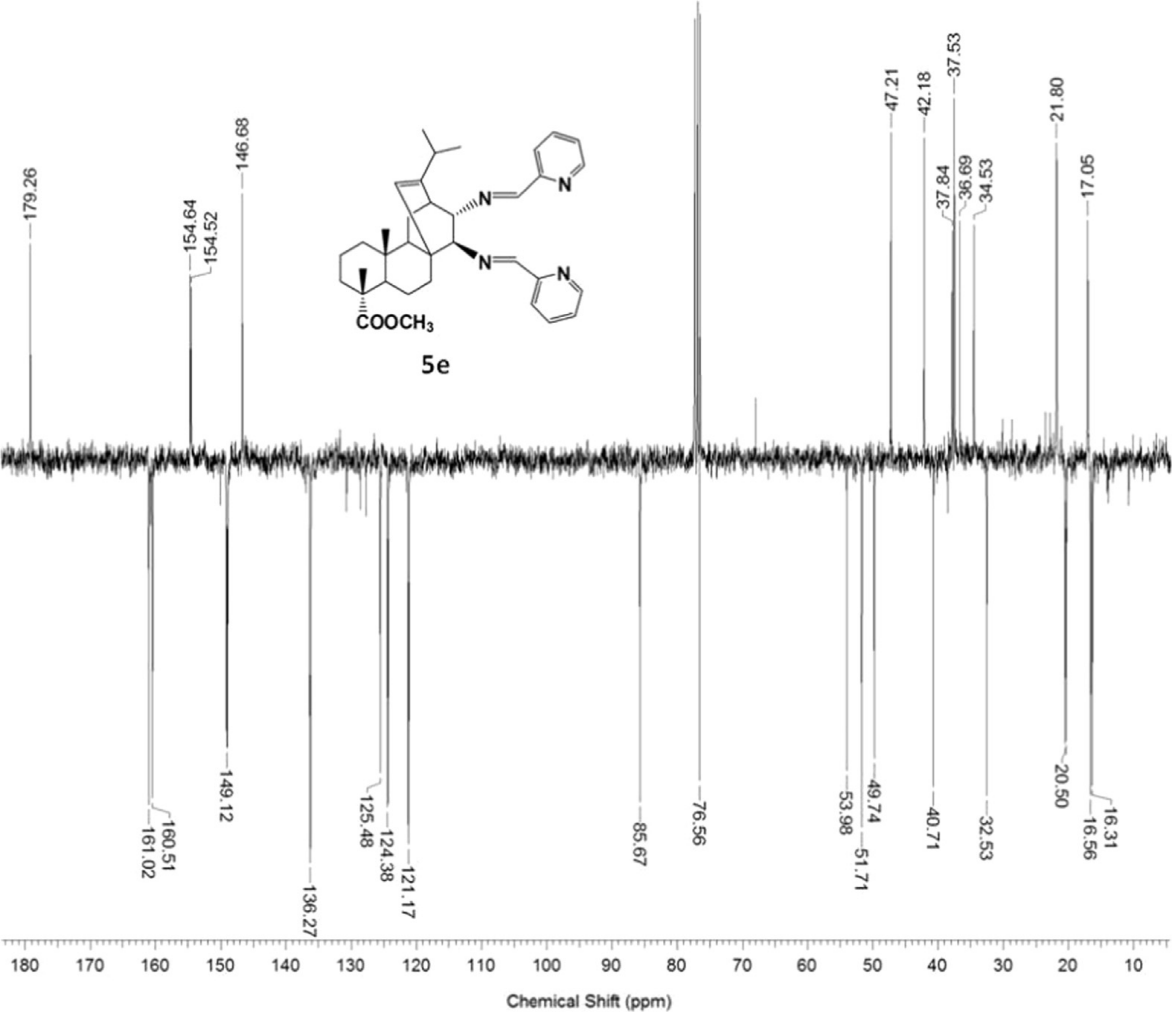
^13^C NMR spectrum of compound **5e**.

**Fig. 5e-3:**
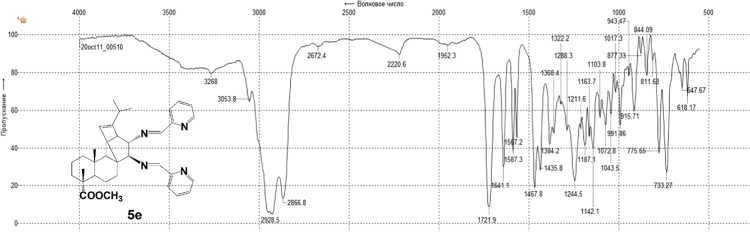
IR spectrum of compound **5e**.

#### 3.5.6 13-isopropyl-17,18-dinor-atis-13-ene-15β,16α-di(thioph-2-ylidenamino)-4-carboxylic acid methyl ester 5f

^1^H, ^13^C NMR and IR spectra of the compound **5f** are presented in Fig. 5f-1, Fig. 5f-2, Fig. 5f-3.

**Fig. 5f-1:**
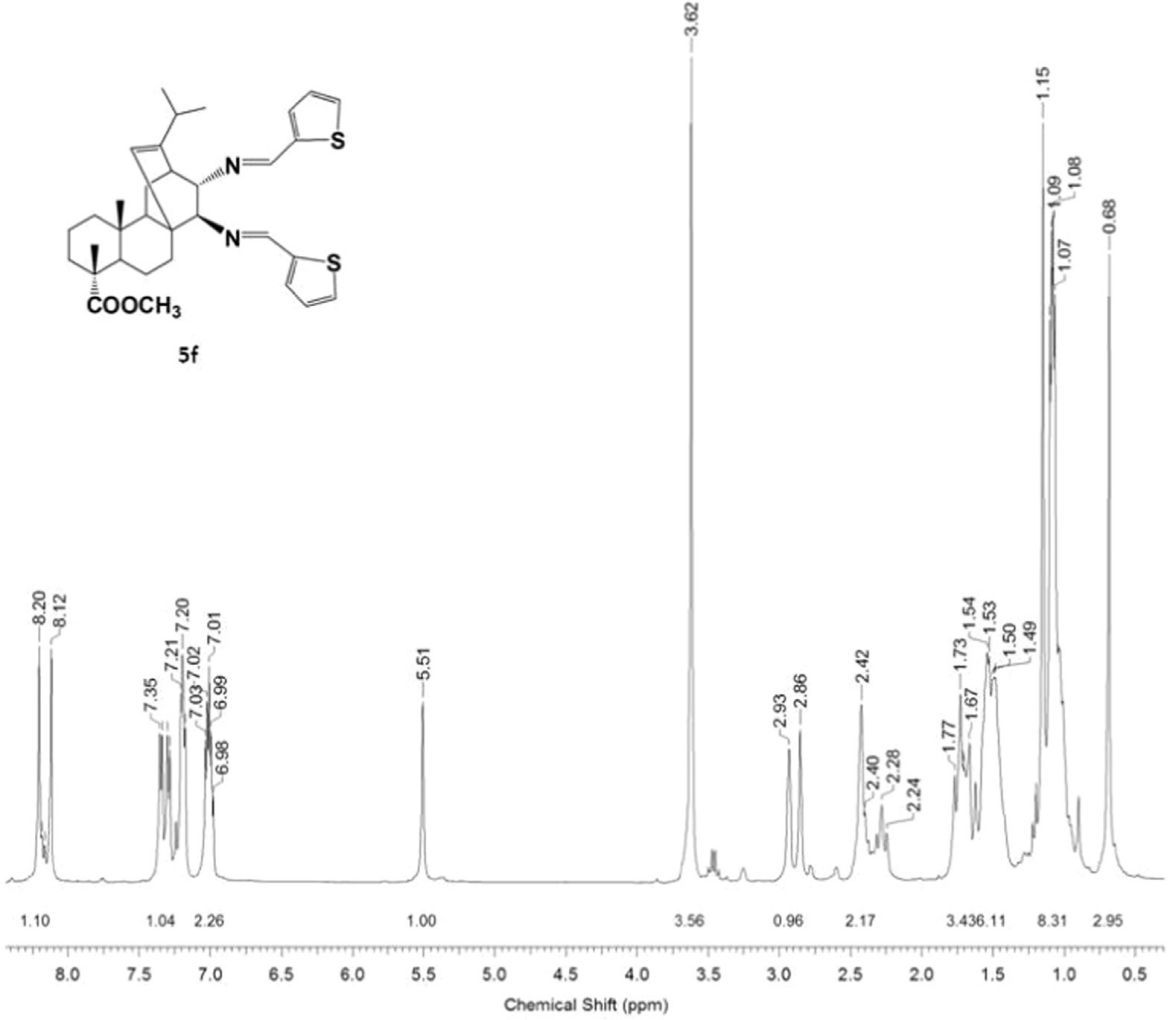
^1^H NMR spectrum of compound **5f**.

**Fig. 5f-2:**
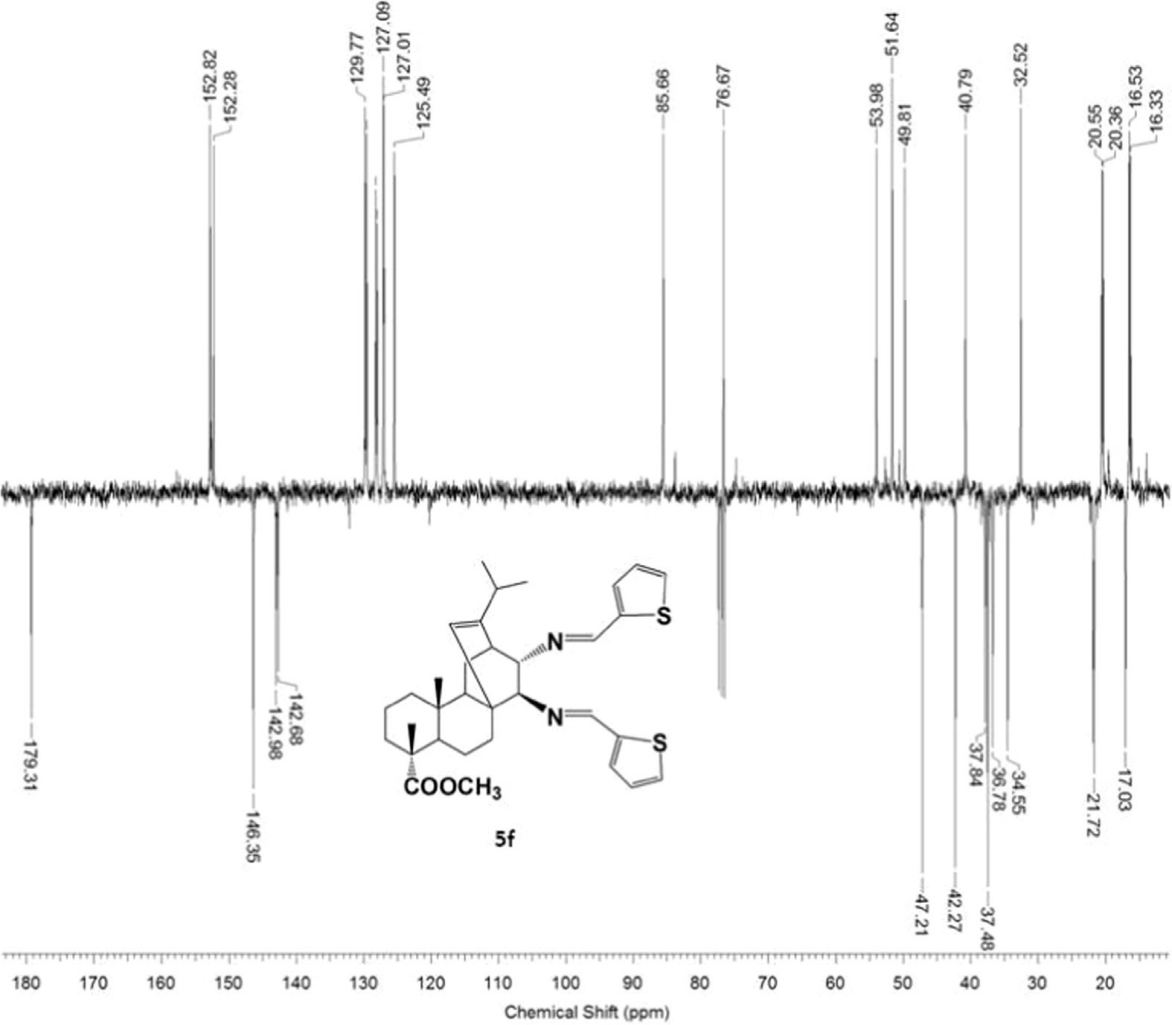
^13^C NMR spectrum of compound **5f**.

**Fig. 5f-3:**
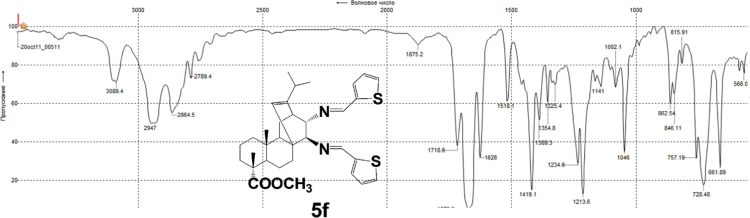
IR spectrum of compound **5f**.

### 3.6 Aminophenols 6a-f

#### 3.6.1 13-isopropyl-17,18-dinor-atis-13-ene-15β,16α-di[(2-hydroxybenzyl)amino]-4-carboxylic acid methyl ester 6a

^1^H, ^13^C NMR and IR spectra of the compound **6a** are presented in Fig. 6a-1, Fig. 6a-2, Fig. 6a-3.

**Fig. 6a-1:**
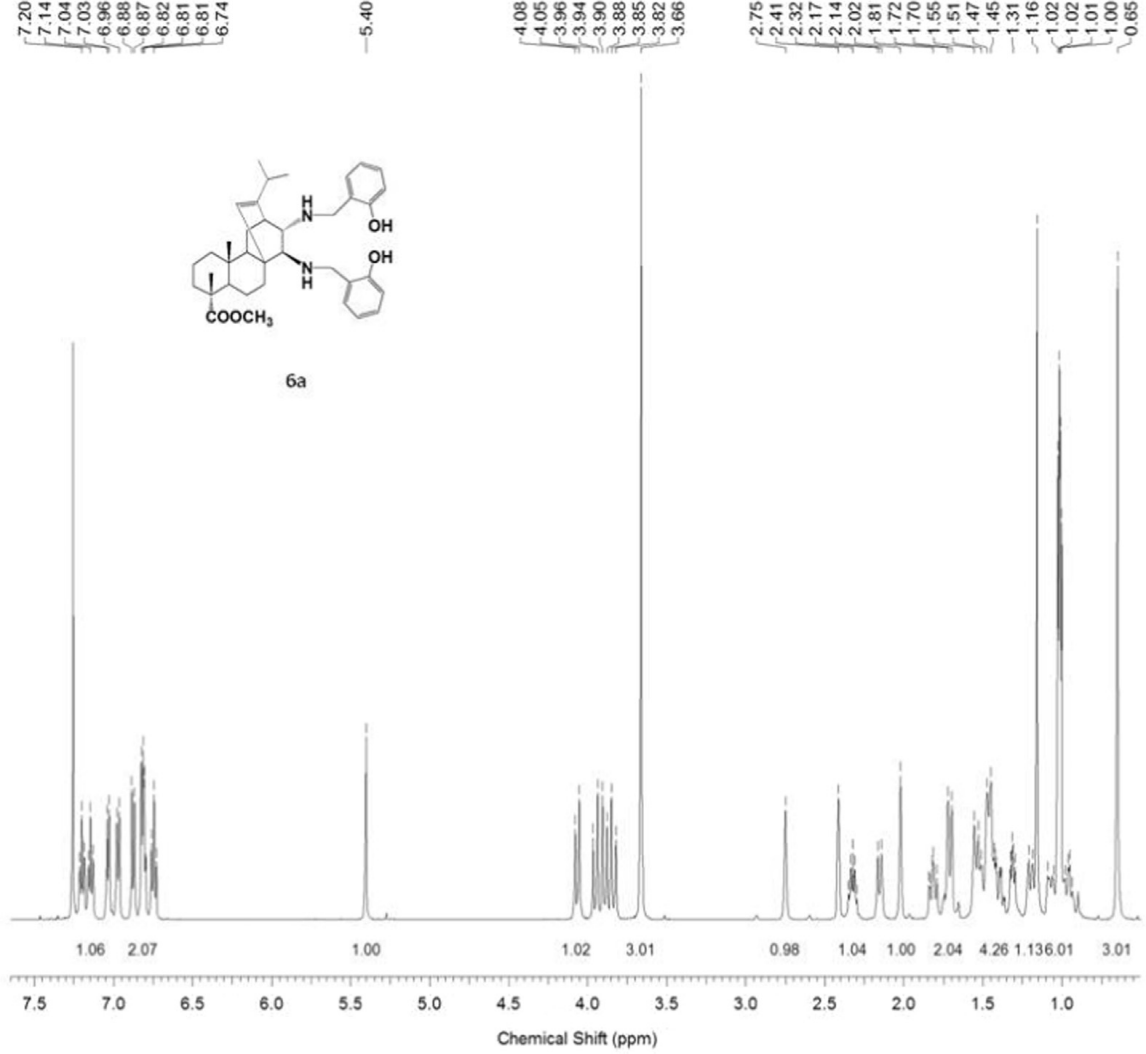
^1^H NMR spectrum of compound **6a**.

**Fig. 6a-2:**
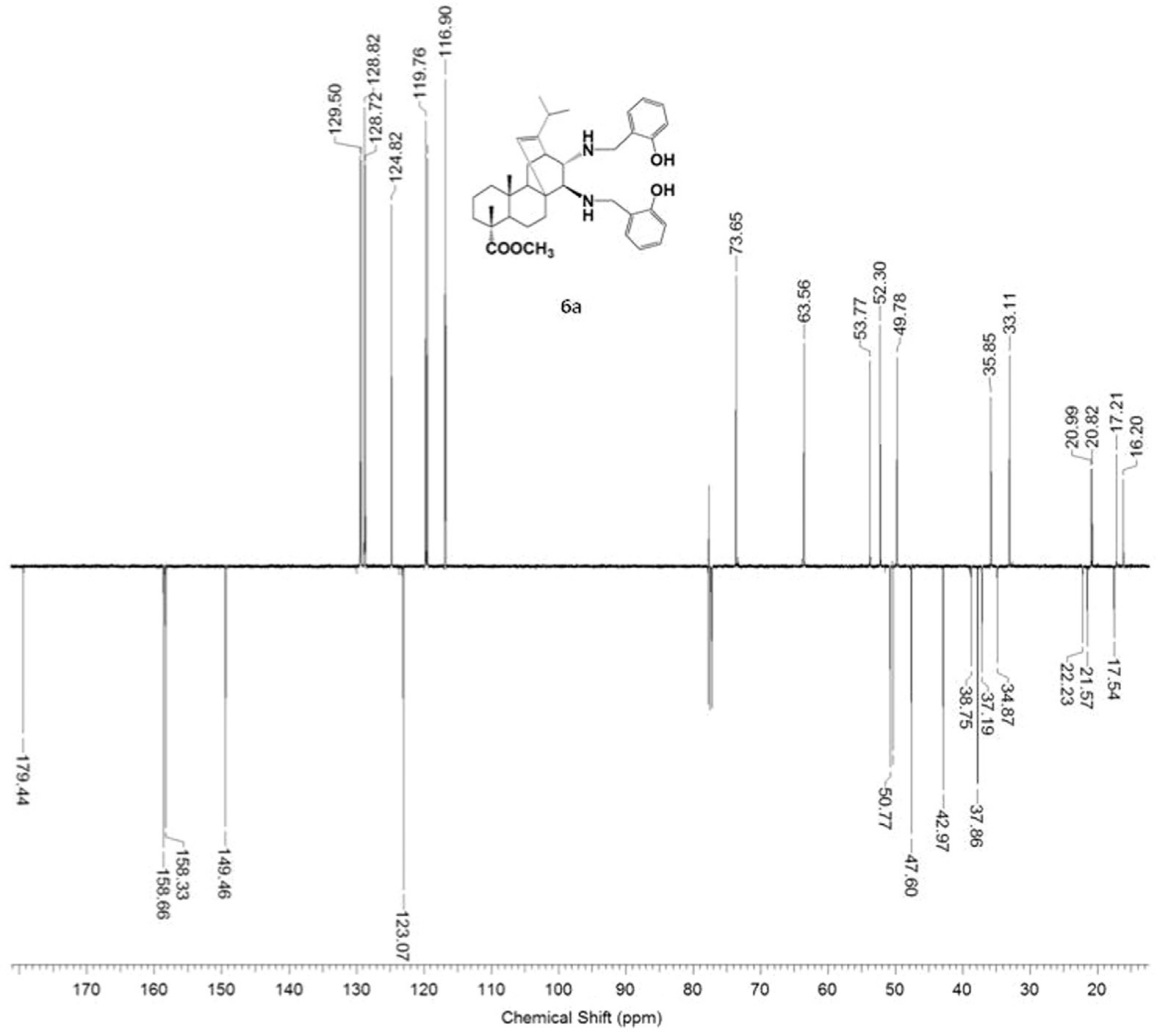
^13^C NMR spectrum of compound **6a**.

**Fig. 6a-3:**
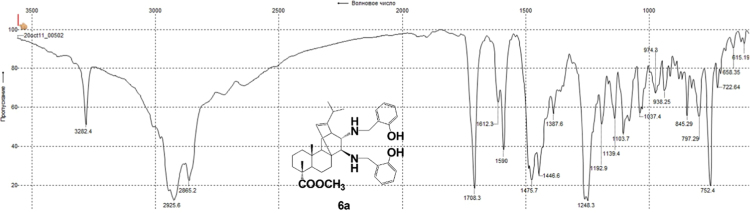
IR spectrum of compound **6a**.

#### 3.6.2 13-isopropyl-17,18-dinor-atis-13-ene-15β,16α-di[(2-hydroxy-3-methoxybenzyl)amino]-4-carboxylic acid methyl ester 6b

^1^H, ^13^C NMR and IR spectra of the compound **6b** are presented in Fig. 6b-1, Fig. 6b-2, Fig. 6b-3.

**Fig. 6b-1:**
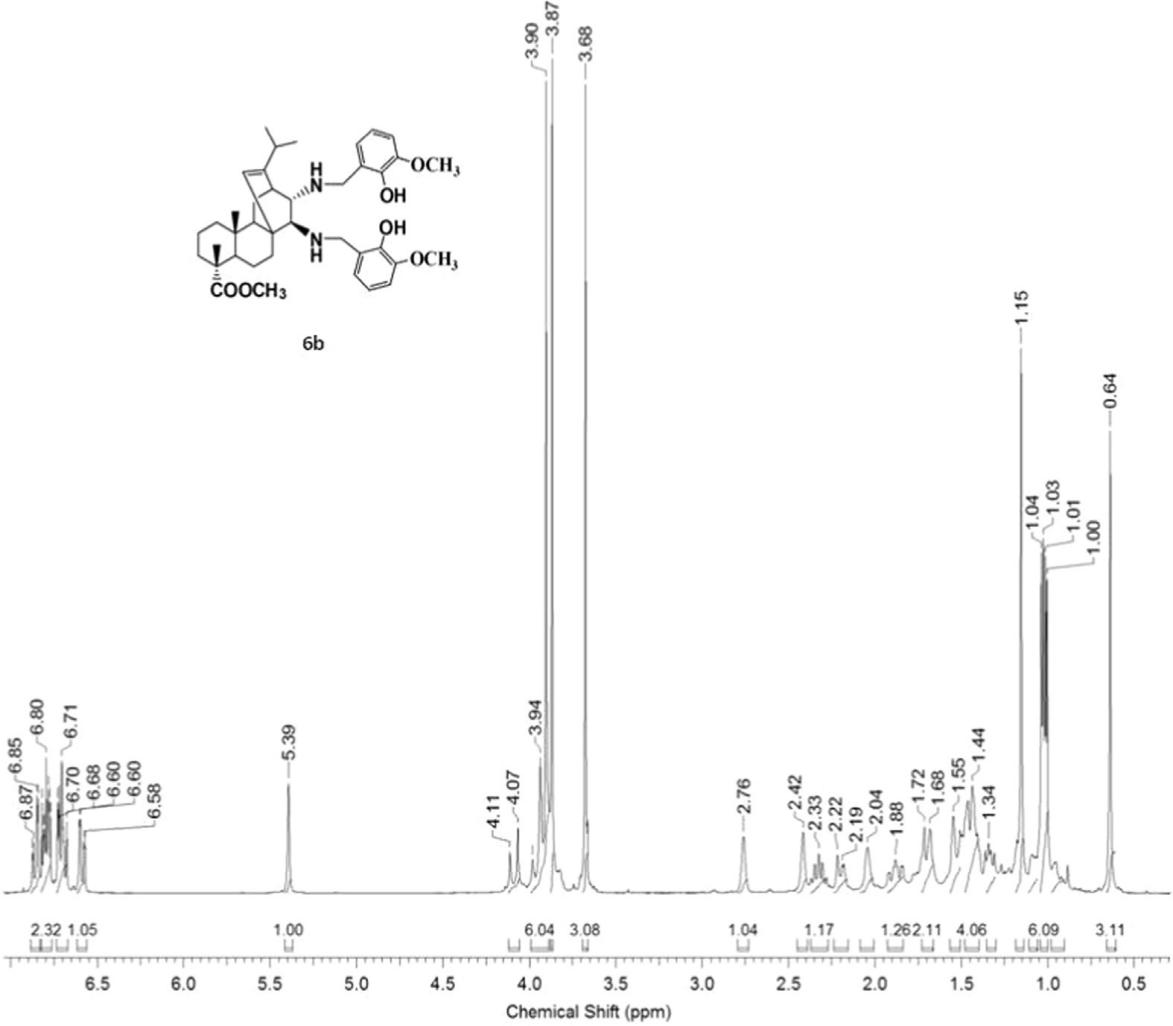
^1^H NMR spectrum of compound **6b**.

**Fig. 6b-2:**
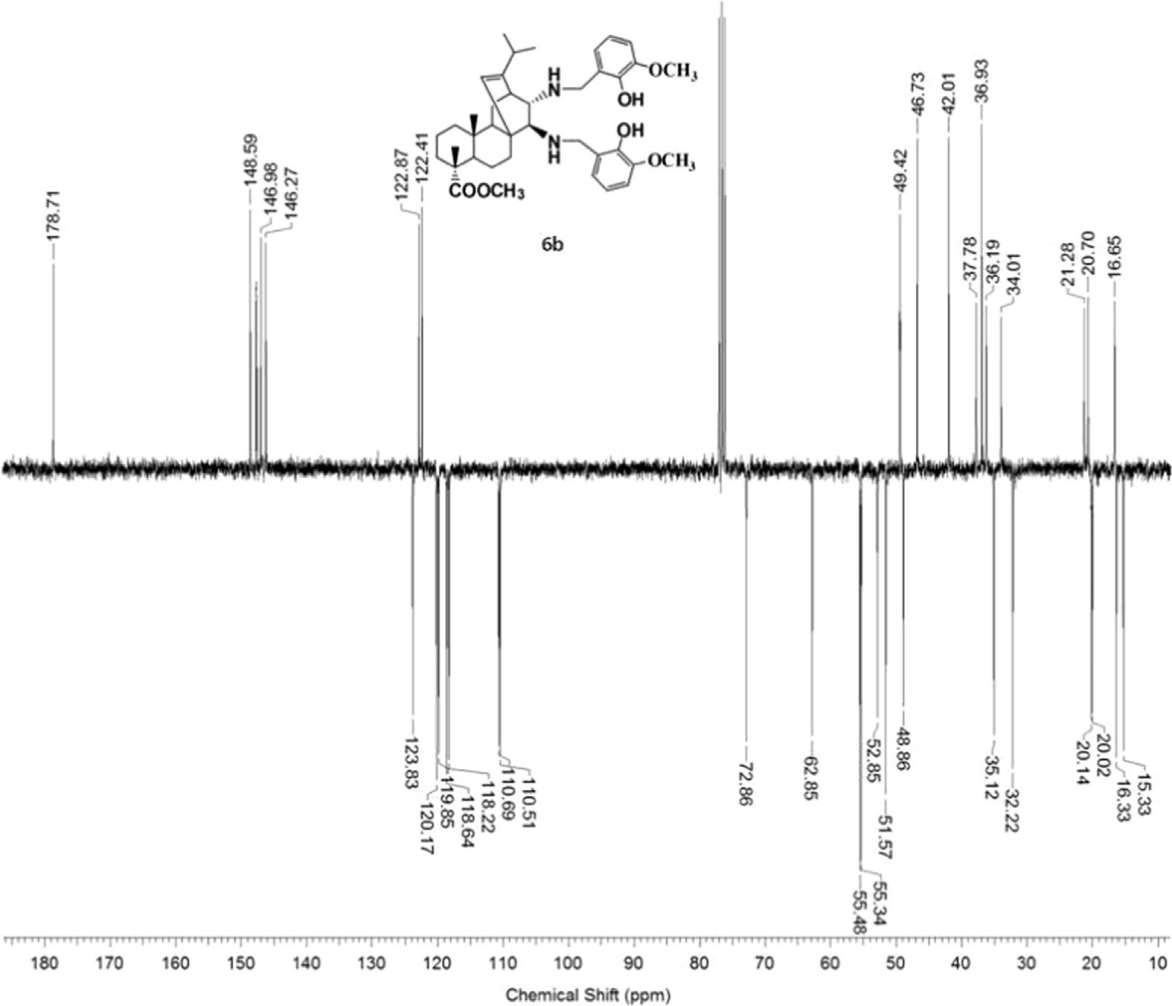
^13^C NMR spectrum of compound **6b**.

**Fig. 6b-3:**
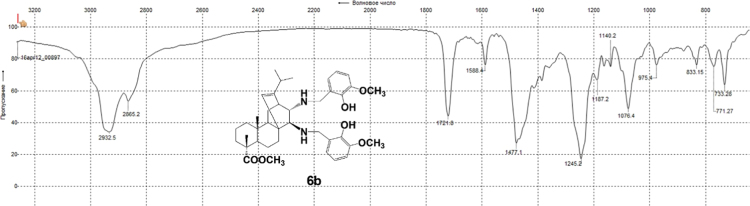
IR spectrum of compound **6b**.

#### 3.6.3 13-isopropyl-17,18-dinor-atis-13-ene-15β,16α-di[(2-hydroxy-3,5-di-tert-butylbenzyl)amino]-4-carboxylic acid methyl ester 6c

^1^H, ^13^C NMR and IR spectra of the compound **6c** are presented in Fig. 6c-1, Fig. 6c-2, Fig. 6c-3.

**Fig. 6c-1:**
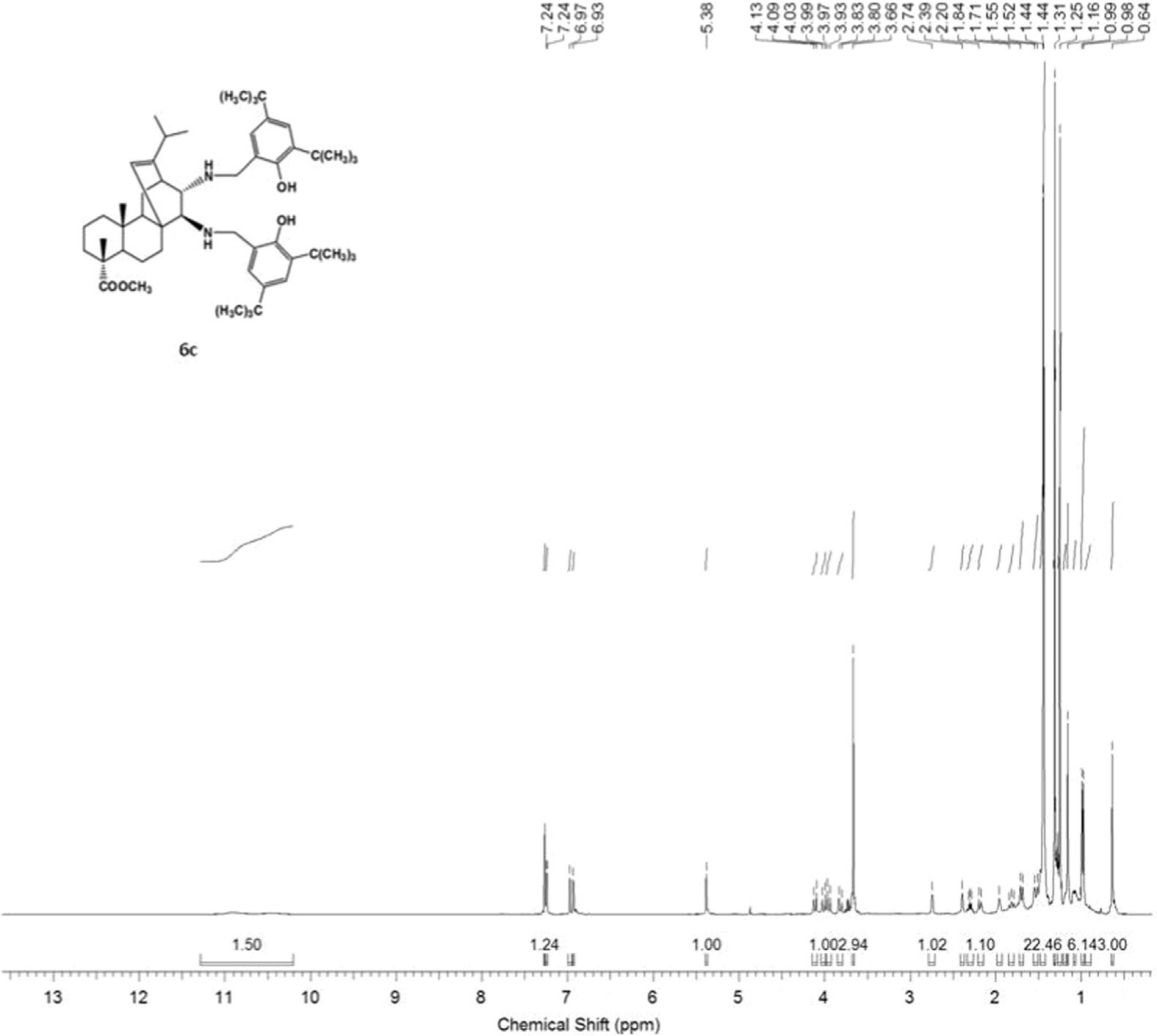
^1^H NMR spectrum of compound **6c**.

**Fig. 6c-2:**
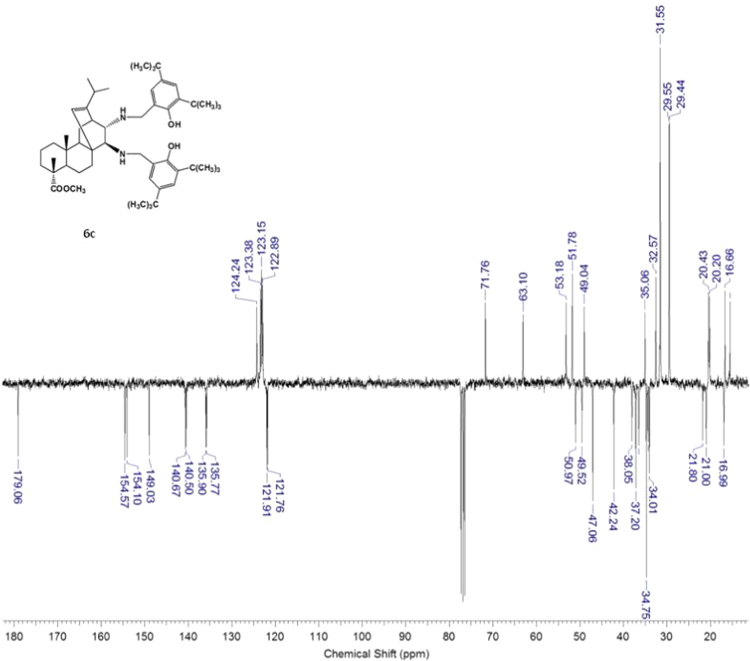
^13^C NMR spectrum of compound **6c**.

**Fig. 6c-3:**
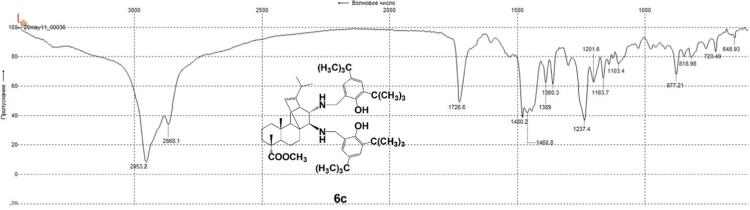
IR spectrum of compound **6c**.

#### 3.6.4 13-isopropyl-17,18-dinor-atis-13-ene-15β,16α-di[(2-hydroxynaphthalen-1-yl)methylamino]-4-carboxylic acid methyl ester 6d

^1^H, ^13^C NMR and IR spectra of the compound **6d** are presented in Fig. 6d-1, Fig. 6d-2, Fig. 6d-3.

**Fig. 6d-1:**
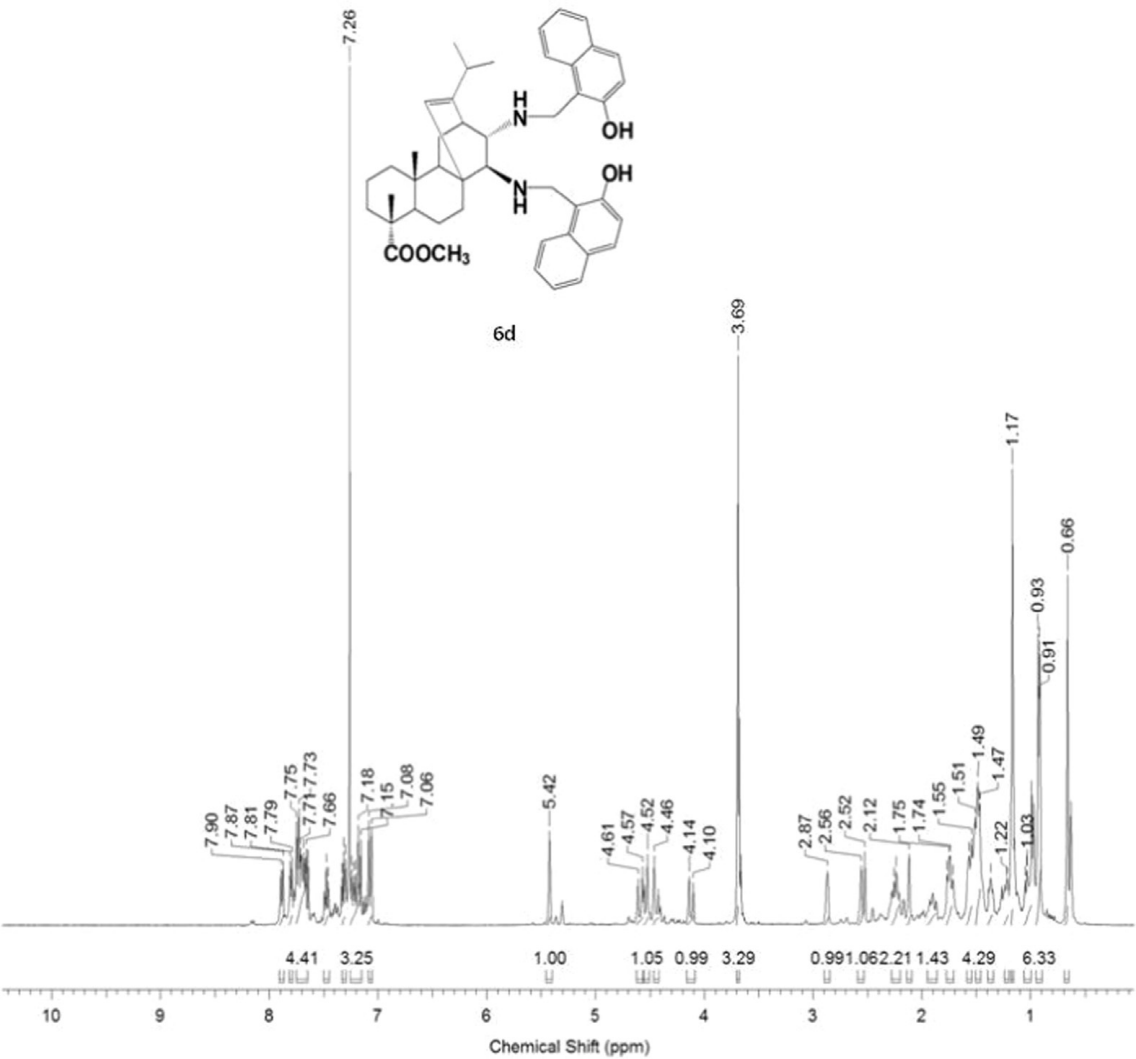
^1^H NMR spectrum of compound **6d**.

**Fig. 6d-2:**
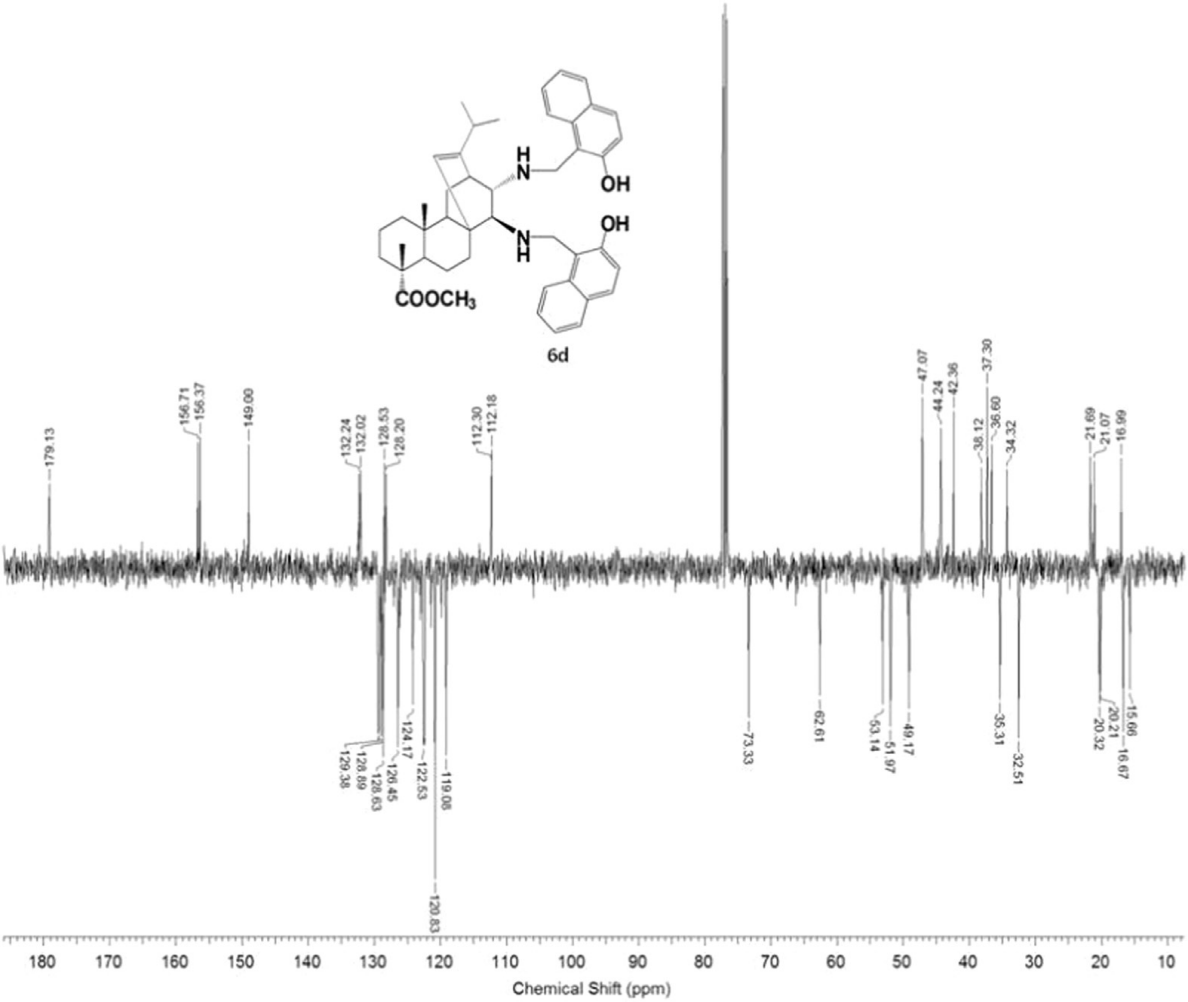
^13^C NMR spectrum of compound **6d**.

**Fig. 6d-3:**
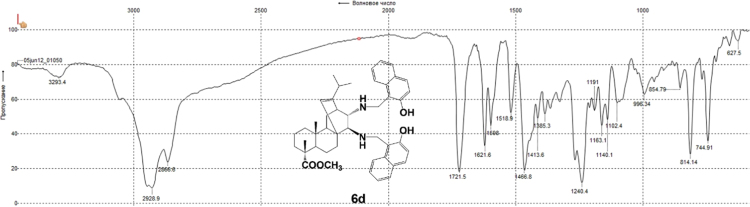
IR spectrum of compound **6d**.

#### 3.6.5 13-isopropyl-17,18-dinor-atis-13-ene-15β,16α-di[(2-pyridylmethyl)amino]-4-carboxylic acid methyl ester 6e

^1^H, ^13^C NMR and IR spectra of the compound **6e** are presented in Fig. 6e-1, Fig. 6e-2, Fig. 6e-3.

**Fig. 6e-1:**
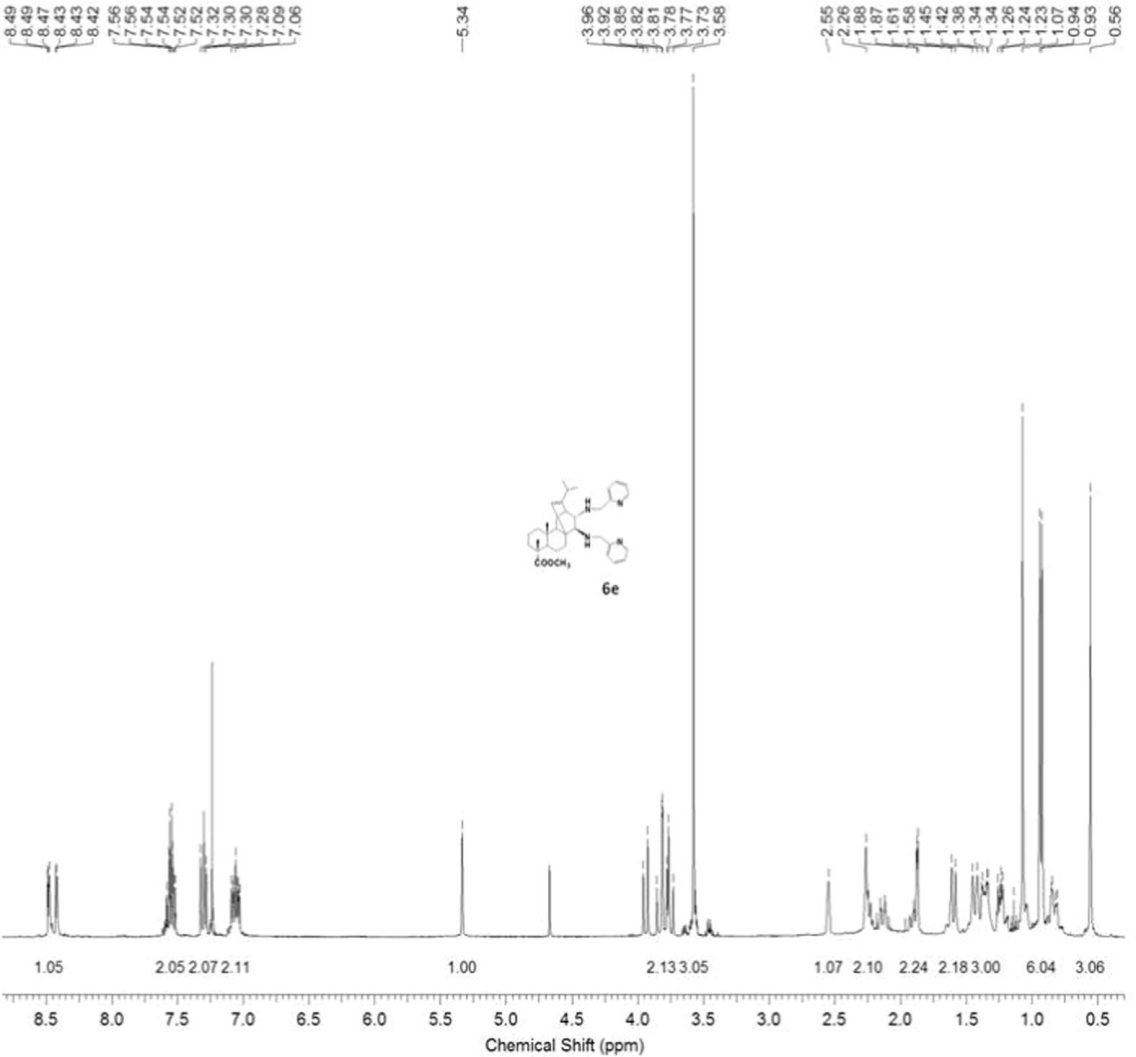
^1^H NMR spectrum of compound **6e**.

**Fig. 6e-2:**
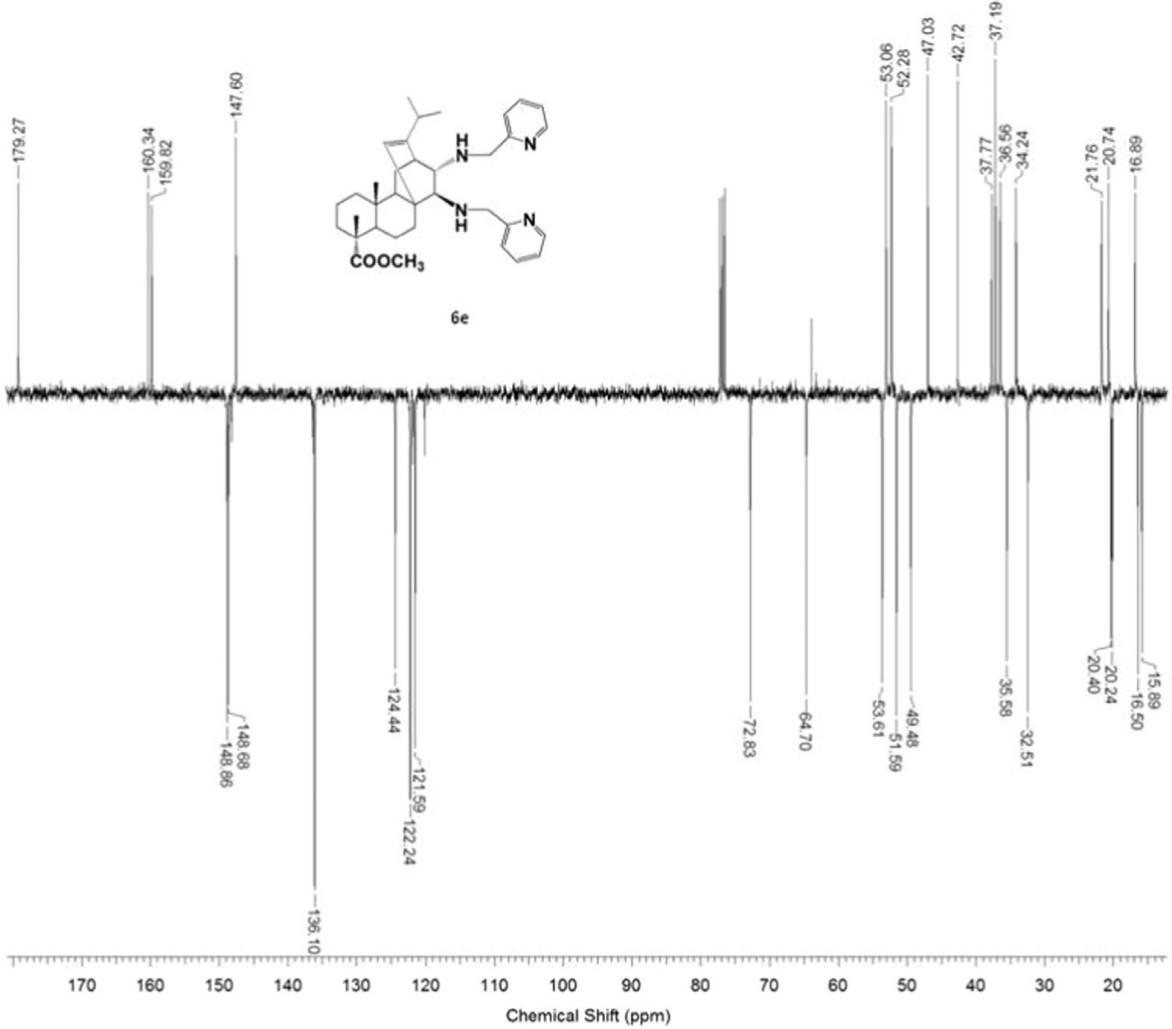
^13^C NMR spectrum of compound **6e**.

**Fig. 6e-3:**
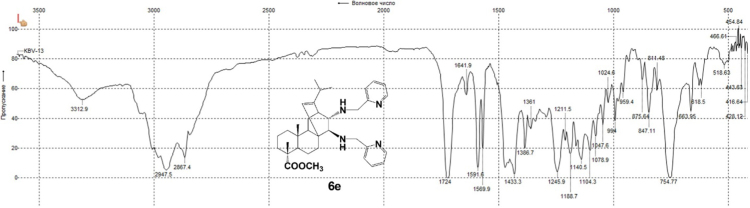
IR spectrum of compound **6e**.

#### 3.6.6 13-isopropyl-17,18-dinor-atis-13-ene-15β,16α-di(((thiophen-2-yl)-methyl)amino)-4-carboxylic acid methyl ester 6f

^1^H, ^13^C NMR and IR spectra of the compound **6f** are presented in Fig. 6f-1, Fig. 6f-2, Fig. 6f-3.

**Fig. 6f-1:**
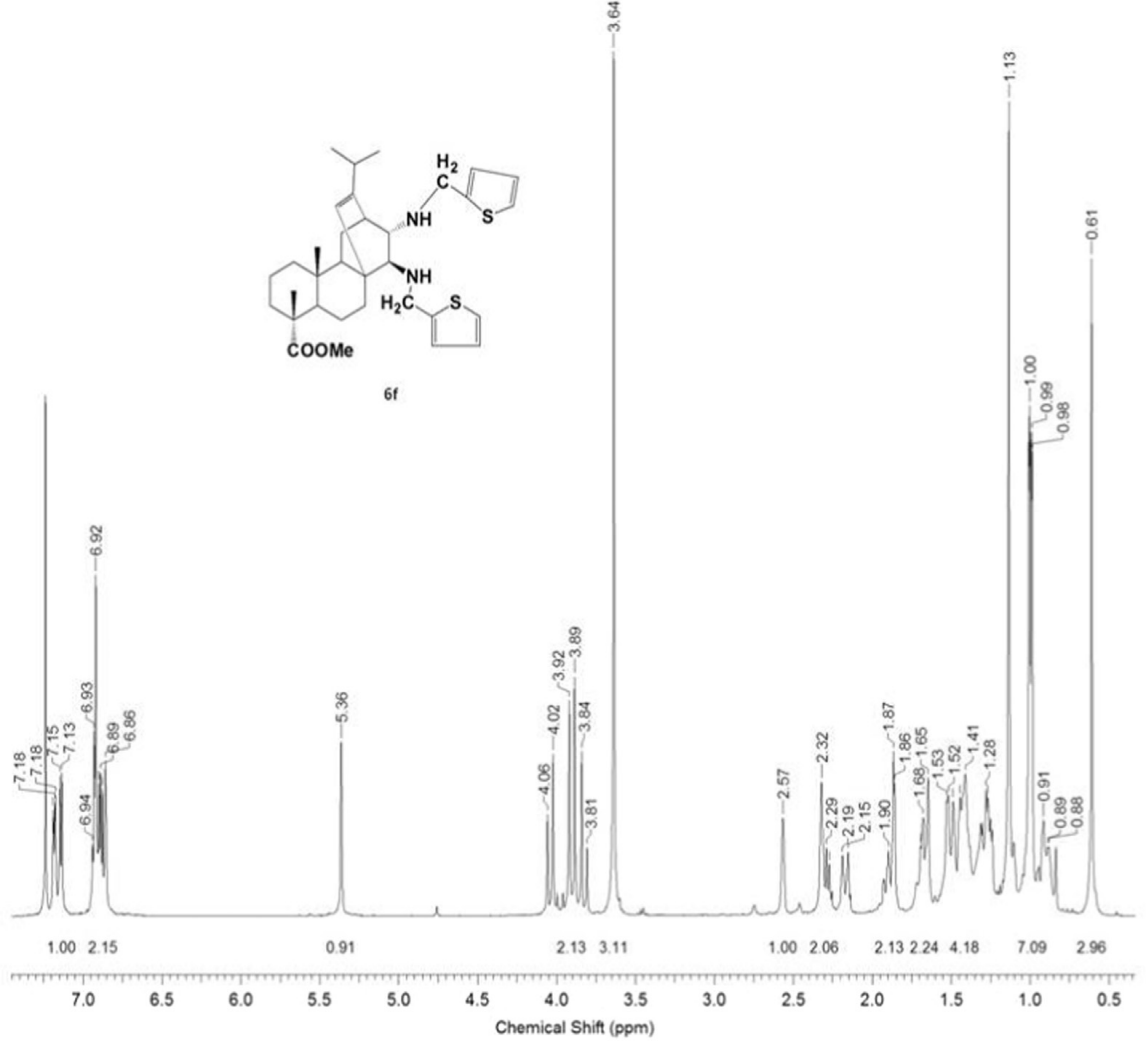
^1^H NMR spectrum of compound **6f**.

**Fig. 6f-2:**
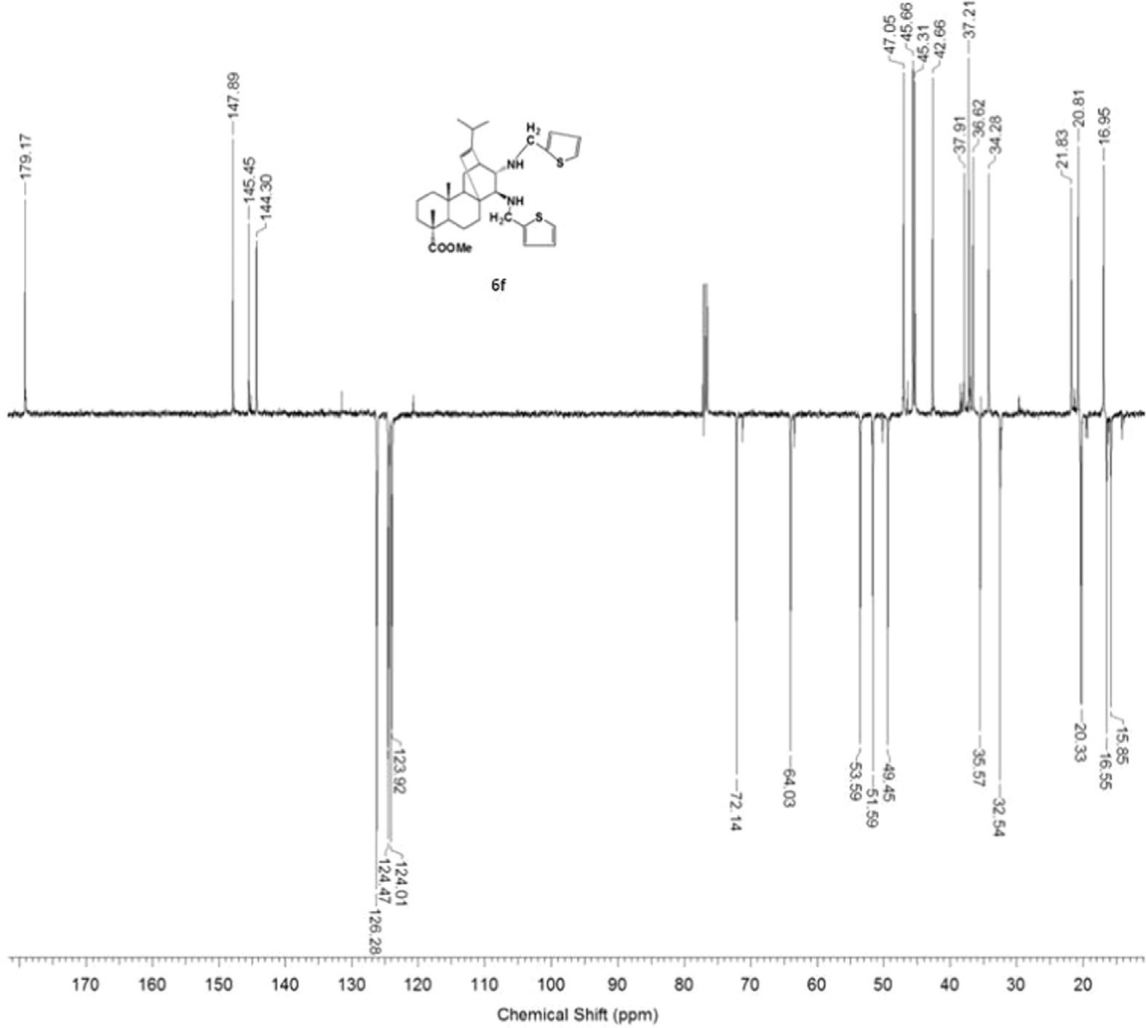
^13^C NMR spectrum of compound **6f**.

**Fig. 6f-3:**
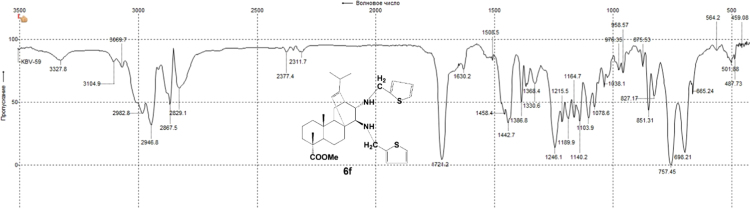
IR spectrum of compound **6f**.
